# Evolutionary history of Methyltransferase 1 genes in hexaploid wheat

**DOI:** 10.1186/1471-2164-15-922

**Published:** 2014-10-23

**Authors:** Mélanie Thomas, Lise Pingault, Axel Poulet, Jorge Duarte, Mickaël Throude, Sébastien Faure, Jean-Philippe Pichon, Etienne Paux, Aline Valeska Probst, Christophe Tatout

**Affiliations:** UMR CNRS 6293 INSERM U 1103 Clermont Université, Genetics Reproduction and Development (GReD), 24 avenue des Landais, BP80026, 63171 Aubière Cedex, France; BIOGEMMA, route d’Ennezat, Centre de Recherche de Chappes, CS 90126, 63720 Chappes, France; UMR INRA 1095 Blaise Pascal University, Genetics Diversity & Ecophysiology of Cereals (GDEC), Clermont-Ferrand – Theix, 5 chemin de Beaulieu, 63039 Clermont-Ferrand Cedex 2, France

**Keywords:** DNA methylation, Evolution, Genome dynamics, CG-rich isochores

## Abstract

**Background:**

Plant and animal methyltransferases are key enzymes involved in DNA methylation at cytosine residues, required for gene expression control and genome stability. Taking advantage of the new sequence surveys of the wheat genome recently released by the International Wheat Genome Sequencing Consortium, we identified and characterized *MET1* genes in the hexaploid wheat *Triticum aestivum* (*TaMET1*).

**Results:**

Nine *TaMET1* genes were identified and mapped on homoeologous chromosome groups 2A/2B/2D, 5A/5B/5D and 7A/7B/7D. Synteny analysis and evolution rates suggest that the genome organization of *TaMET1* genes results from a whole genome duplication shared within the grass family, and a second gene duplication, which occurred specifically in the *Triticeae* tribe prior to the speciation of diploid wheat. Higher expression levels were observed for *TaMET1* homoeologous group 2 genes compared to group 5 and 7, indicating that group 2 homoeologous genes are predominant at the transcriptional level, while group 5 evolved into pseudogenes. We show the connection between low expression levels, elevated evolution rates and unexpected enrichment in CG-dinucleotides (CG-rich isochores) at putative promoter regions of homoeologous group 5 and 7, but not of group 2 *TaMET1* genes. Bisulfite sequencing reveals that these CG-rich isochores are highly methylated in a CG context, which is the expected target of *TaMET1*.

**Conclusions:**

We retraced the evolutionary history of *MET1* genes in wheat, explaining the predominance of group 2 homoeologous genes and suggest CG-DNA methylation as one of the mechanisms involved in wheat genome dynamics.

**Electronic supplementary material:**

The online version of this article (doi:10.1186/1471-2164-15-922) contains supplementary material, which is available to authorized users.

## Background

*Triticum aestivum* (hexaploid wheat or bread wheat) is one of the most important cultivated species in the world and it has been subject of intense research. Investigations of its genome structure led to the discovery of its highly dynamic nature during evolution. Using fossil records and phylogenetic studies, its evolution was traced from ancestral diploid species proposed to originate 50–77 million years ago (MYa) [1]. Indeed, bread wheat is a hexaploid species made of three homoeologous genomes called A, B and D which derived from different diploid species. These are proposed to be *Triticum urartu* (2n = 2× = 14 chromosomes, AA) and a diploid species related to *Aegilops speltoides* (2n = 2× = 14, BB) which gave rise some 0.5 ~ 0.6 MYa ago to *Triticum durum* (2n = 4× = 28 chromosomes, AABB). About 8,000 years ago, hybridization occurred between *Triticum durum* and *Aegilops tauschii* (2n = 2× = 14 chomosomes, DD) and yielded *Triticum aestivum* (2n = 6× = 42 chromosomes, AABBDD), the hexaploid wheat [2]. This means that every single gene is expected to be found in triplicate. The genome structure, organized in homoeologous genomes A, B and D, has to be maintained through cell division, a function which is ensured by the *Ph1* suppressor locus. The *Ph1* locus restricts homoeologous recombination and permits proper chromosome segregation in a hexaploid context through mitosis and meiosis [3].

Complementary approaches known as comparative genomics [4] at the genome-level (synteny) or the chromosome level (micro-synteny) were used to predict the genome structure of wheat in comparison to sequenced diploid species such as rice [5, 6], sorghum [7], maize [8], brachypodium [9] and more recently barley [10]. Recent syntenic studies proposed that the ancestral genome of grass species was a diploid species organized in five chromosomes (2n = 2× = 10 chromosomes) [11]. From this initial chromosome organization, the ancestral diploid genome was duplicated through Whole-Genome Duplication (WGD) then fragmented giving rise to an intermediate ancestor with 2n = 2× = 24 chromosomes [11]. This genomic structure has been well conserved in rice (2n = 2× = 24 chromosomes) while it evolved to 2n = 2× = 14 through chromosome rearrangements in diploid wheat. Although WGD is expected to have had a large impact on wheat genome evolution it is not the only mode of genome rearrangement. Indeed, duplication of large chromosomal regions (segmental duplication), duplication at the gene level or tandem duplications have also occurred in the course of evolution [12]. Furthermore, it is now well established that wheat genome organization has been largely influenced by transposable element mobilization [13]. Most of the mechanisms described above increase genome size and lead to an elevated gene copy number. However, much less is known about reverse mechanisms, which reduce genome size to restore a diploid situation and reform single copy gene states. Indeed, early studies in *Saccharomyces cerevisiae* indicate that only 12% of the duplicate pairs remain after WGD suggesting that an extensive gene loss occurs after WGD [14]. In flowering plants, a fraction of single-copy genes were recently investigated and new hypotheses were suggested [15]: basically, after duplication, genes within one of the duplicated segments tend to be lost through small deletions while most genes are retained within the second segment, a mechanism known as fractionation bias [16]. Another difference occurring after duplication between two genomic segments is known as genome dominance during which one of the two segments shows higher expression levels than the other [16]. Data from maize and brassica further indicate that both gene fractionation, leading to extensive gene loss, and genome dominance are occurring simultaneously keeping the expression of the retained genes at elevated levels [16, 17]. Hexaploid wheat does not show an overall genome-wide transcriptional dominance of A, B or D subgenomes although some homoeologous genes can adopt a specific expression pattern [18]. All these recent outcomes reveal important genome dynamics, which affect genome size or organization and alter gene expression. However, mechanisms implicated in these phenomena remain largely hypothetical, although epigenetic mechanisms have been suggested to provide means to induce asymmetric levels of expression between the two duplicated fragments prior to gene fractionation [16].

Although our knowledge about the hexaploid wheat genome structure is increasing, it remains challenging to decipher every step leading to its large genome size of about 16–17 Gb, which includes up to 80% of repeated sequences [13]. In polyploid genomes like cotton, rapeseed or wheat, several studies suggested the importance of epigenetic mechanisms in maintaining genome structure and chromatin stability as well as in regulating gene expression after hybridization and polyploidization [19, 20]. One of these epigenetic mechanisms is DNA methylation, which takes place at the carbon-5 cytosine residues in CG, CHG and CHH (where H = A, T or C) contexts [21]. Loss of DNA methylation causes reactivation of silenced transposable elements [22] and the expression of certain genes, such as *FWA*, a gene involved in flowering [23, 24]. DNA methylation is also known to affect crossover rate and meiotic recombination [25].

We wanted to reconstruct the evolutionary history of the hexaploid wheat species *Triticum aestivum* using the example of *MET1*, a gene encoding METHYLTRANSFERASE 1 (MET1), responsible for DNA methylation maintenance in the CG context. *MET1* is a gene of particular importance for genome maintenance in many organisms, which we hypothesize will be a crucial component of epigenetic mechanisms controlling transposable elements that in wheat make up to 80% of the genome. To date *MET1* gene function have been described in several plant species including Arabidopsis [26], maize [27], rice [28] and brassica [29] but not in wheat. We identified *MET1* genes in hexaploid wheat (*TaMET1*). Nine copies of *TaMET1* are organized in three paralogous groups at chromosome 2, 5 and 7 suggesting that the genomic regions including *MET1* genes were subjected to two duplication events prior to the emergence of hexaploid wheat. Considering *TaMET1* genomic regions specifically, we confirmed that the first gene duplication was part of a WGD common to all grass species and that the second duplication occurred through gene duplication specific to the *Triticeae* tribe. Expression profiles of the different *MET1* gene copies, estimation of their evolution rates, CG enrichment and methylation profiles highlight the predominance of group 2 homoeologous genes at the transcript level. Our results exemplify the high dynamics of genome evolution in the course of the evolutionary history of bread wheat and suggest the involvement of epigenetic mechanisms in these processes.

## Results

### Hexaploid wheat contains nine *TaMET1*loci

**Figure 1:**
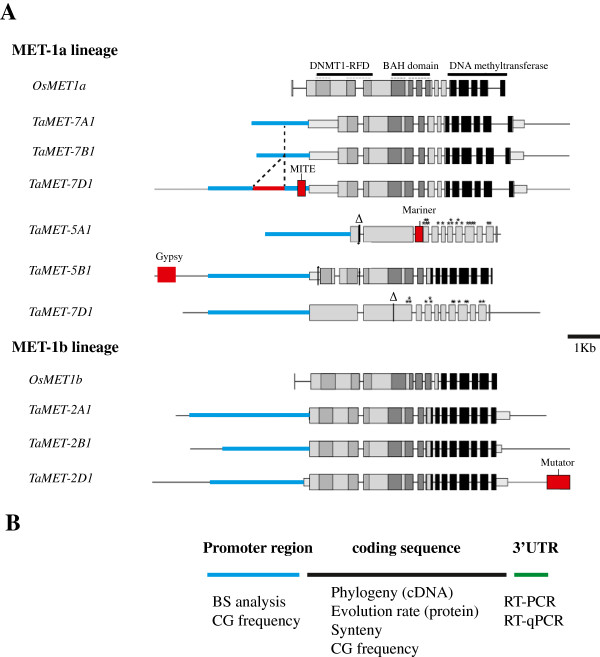
**Gene structures of** ***TaMET1*** **genes. A)** The two *MET1* genes from rice (*OsMET1a*; [GenBank: Os03g58400] and *OsMET1b*; [GenBank: Os07g08500]) were used as a reference to define two MET1 lineages (hereafter called MET-1a and MET-1b lineages). *OsMET1a* and *OsMET1b* are shown at the top of each lineage. Gene structures and splice junctions organize *TaMET1* genes in 11 exons (black and grey boxes) and 10 introns (horizontal lines). The three distinct protein domains identified are indicated at the top of the figure: DNMT1-RFD (light grey), BAH-domain (dark grey) and DNA methyltransferase (black). *TaMET1* genes from chromosome 2 and 7 as well as chromosome 5B are predicted to yield a full-length MET1 protein while 5A and 5D contain stop codons (*) and deletions (Δ). 5′ and 3′UTRs are indicated as smaller boxes. Potential promoter regions were defined as the ~2-3 kb region upstream of the coding sequences and are indicated as a thicker blue line. Unknown sequence insertion (*TaMET-7D1*) is indicated as a red line and transposable element insertion of Stowaway, Gypsy, Mariner and Mutator are indicated as red boxes. **B)** Methods used in this study.

In order to determine the number and complete sequence of *TaMET1* genes, we chose a genomic strategy based on a combination of sequence capture and *in silico* mining of available wheat genome sequences. In order to define probes on the sequence capture microarray, *TaMET1*-expressed tags (ESTs) were identified in wheat databases. Eight ESTs were retrieved from public and private libraries. The alignment of these ESTs with the rice and brachypodium *MET1* genes showed that these ESTs mapped to the 3′end of *TaMET1* genes. Two *TaMET1* ESTs as well as two brachypodium *MET1* genomic fragments were selected and used to design probes for sequence capture (see Methods). Two successive runs of sequence capture yielded 8,184 reads specific to *TaMET1* genes. Genomic fragments were then assembled *de novo* using gsAssembler in six large contigs corresponding to six putative *TaMET1* genes. However some reads remained impossible to assemble and could not be included within the six large contigs suggesting the possible existence of additional copies of *TaMET1*. In parallel, the draft genome assembly of the wheat genome released by Brenchley and collaborators [30] was mined for *TaMET1* genes. However, no full-length sequences corresponding to *TaMET1* genes were present in the dataset. Taking advantage of the recent release of sequence surveys from the International Wheat Genome Sequencing Consortium (IWGSC) (http://www.wheatgenome.org/) that were produced from sorted chromosome arms [18], BLASTn analyses against each chromosome arm were performed using rice and brachypodium *MET1* genes. Eventually nine *MET1* copies were identified and assigned to chromosomes 2A/2B/2D, 5A/5B/5D and 7A/7B/7D. For simplicity, homoeologous chromosomes A, B and D will be collectively referred to as a “homoeologous group” hereafter. Intron and exon junctions were defined for the nine *TaMET1* genes according to rice and brachypodium *MET1* genes and subsequently validated by RNA-seq analysis (see below). Protein domains were then predicted using the Pfam database. Three major protein domains were identified that include DNMT1-RFD (Cytosine specific DNA methyltransferase replication foci domain), BAH (Bromo-Adjacent Homology) and the DNA methyltransferase (C5 cytosine specific DNA methylase) domain (Figure 1). Comparison with *MET1* genes from rice orthologs showed an overall conservation of the *TaMET1* genes (Figure 1). Coding sequence analyses revealed that the *TaMET1* genes of chromosome 5A and 5D display deletions and premature stop codons (Figure 1) and if expressed produce truncated proteins missing the DNA methyltransferase domain. *TaMET-5A1* and *5D1* may be considered as pseudogenes, while all the remaining genes are expected to be functional.

### *TaMET1*loci originated from two successive duplication events

**Figure 2:**
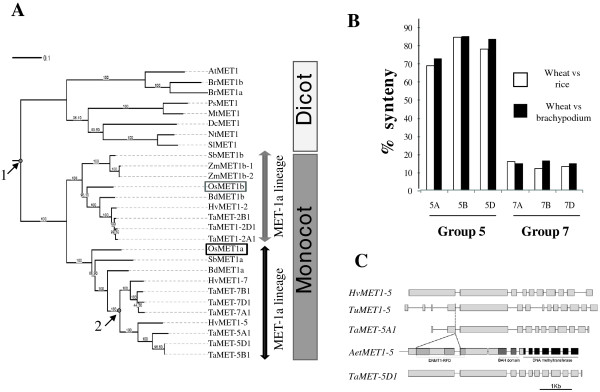
***MET1*** **phylogenetic trees in flowering plants and syntenic conservation. A)** Maximum likelihood (ML) phylogenetic tree. The ML tree was inferred using cDNA sequences from dicot species including *Arabidopsis thaliana* (*AtMET1*: [TAIR:At5g49160]), *Brassica rapa* (*BrMET1a* and *BrMET1b* respectively [GenBank: AB251938 and AB25937]), *Pisum sativum* (*PsMET1*: [GenBank: AF034419]), *Medicago truncatula*: (*MtMET1*: [Phytozome: Medtr6g065580]), *Daucus carota* (*DcMET1*: [GenBank: AF007807]), *Nicotiana tabacum* (*NtMET1*: [Genbank: AB030726]), *Solanum lycopersicum* (*SlMET1*: [GenBank: AJ002140]), and monocot species including *Sorghum bicolor* (*SbMET1a* and *SbMET1b* respectively [Phytozome: Sb01g005084 and Sb02g004680]), *Zea mays* (*ZmMET1b-1* and *ZmMET1b-2* respectively [Phytozome: GRMZM2G333916 and GRMZM2G334041]), *Oriza sativa* (*OsMET1a* and *OsMET1b* respectively: [Genbank AB362510 and AB362511]), *Brachypodium distachyon (BdMET1a* and *BdMET1b* respectively [Phytozome: Bradi1g05380 and Bradi1g55287]), *Hordeum vulgare* (HvMET1-2 and HvMET1-7 respectively*:* [Ensembl Genomes: MLOC_61904.6 and: MLOC_10988.2], *HvMET1-5* predicted from [GenBank: CAJW010043285]) and *Triticum aestivum*: *TaMET-2A1*, *2B1*, *2D1*, *5A1*, *5B1*, *5D1*, *7A1*, *7B1* and *7D1* (this study from IWGSC contigs). Numbers above branches indicate bootstrap values. Two monophyletic groups were defined and called the MET-1a and MET-1b lineages according to the *OsMET1a* and *OsMET1b* genes (indicated in boxes). The two gene duplication events observed in wheat and barley are indicated by an arrow. **B)** Micro-synteny analysis. Micro-synteny was established by BLASTn analysis between genes from brachypodium, rice and IWGSC sequences from wheat chromosomes as described in Methods. Syntenic conservation between rice (in white) and brachypodium (in black) correspond to the number of conserved genes in wheat/number of genes in the syntenic region from brachypodium or rice expressed as percentage. **C)** Gene structures of *MET1* genes at chromosome 5. BLASTn analysis was used to align *MET1* genes from *Hordeum vulgare* (*HvMET1-5*: [GenBank: CAJW010043285]), *Triticum urartu* (*TuMET1-5* [Ensembl Plants: scaffold15783]), *Aegilops tauschii* (*AetMET1-5* [Ensembl Plants: scaffold164515]) and *Triticum aestivum* (*TaMET-5A1* and *TaMET-5D1*) (this study).

In a first attempt to understand the genome rearrangements, which have led to the nine *TaMET1* genes present in the *T. aestivum* genome, we retraced the phylogenetic history of *TaMET1* genes using *MET1* orthologs from monocotyledonous and dicotyledonous species. Two distinct copies of *MET1* (*i.e.* two distinct paralogs) are usually found in monocots such as rice, sorghum and brachypodium species. Phylogenetic analysis suggests that homoeologous *TaMET1* genes from group 2 are orthologous to *OsMET1b* on chromosome 7 while homoeologous *TaMET1* genes from group 5 and 7 are orthologous to *OsMET1a* on chromosome 3 (Figure 2A). Hereafter, these two phylogenetic groups are called MET-1a and MET-1b lineages in respect to the *MET1* genes from rice. The phylogenetic tree suggests that a first duplication event occurred early during monocot speciation resulting in the MET-1a and the MET-1b lineages (Figure 2A). Since these two copies of *MET1* are common to all grass species, the first *TaMET1* duplication is likely to be a consequence of the WGD that took place in all grasses and occurred about 56–73 MYa. The second duplication is shared only within the *Triticea* tribe (barley and wheat in our phylogenetic tree). Since wheat diverged from brachypodium 32–39 MYa and from barley 10–13 MYa [1, 9], this second duplication probably occurred between 32 and 13 MYa. In order to understand if this duplication was the result of segmental or single gene duplication, syntenic relationships between regions surrounding the *TaMET1* genes from chromosome 5 and 7 and their orthologous loci in rice and brachypodium were investigated. For chromosome 5A, 5B and 5D, up to 80% of the genes were conserved, whereas only 10-15% were for group 7, suggesting that a single gene duplication occurred (Figure 2B). This hypothesis is consistent with the evolutionary model of grass genomes [11, 31].

In order to date the duplication event leading to group 5 paralogs, BLASTn analyses were conducted between hexaploid wheat (*Triticum aestivum*), diploid wheat species (*Triticum urartu, Aegilops tauschii*) and barley (*Hordeum vulgare*). *Triticum urartu* (genome A ancestor) shares the same deletion with *TaMET-5A1* while *Aegilops tauschii* (genome D ancestor) and *TaMET-5D1* do not (Figure 2C). It can then be suggested that 5A was already in the process of pseudogenization before polyploidization while 5D pseudogenization occurred in the course of, or after, polyploidization. Consistent with this hypothesis, 5A displays a more pronounced gene structure alteration than 5D (large deletion and numerous stop codons; see also Figure 1).

### *TaMET1*genes display distinctive evolution rates

We then investigated the putative functional differences between the nine *TaMET1* genes by evaluation of the evolution rate, which is a good indicator for the biological function of a given gene [32]. We chose the codon substitution model to estimate the rate of synonymous (dS) and non-synonymous (dN) substitutions and computed the dN/dS ratio as evolution rate (ω) [33]. In this model, for genes with a significant biological function undergoing purifying selection non-synonymous mutations are expected to be kept at a low level whereas synonymous mutations accumulate randomly according to the neutral theory of evolution [33].

**Figure 3:**
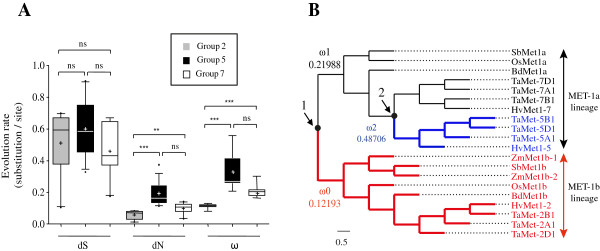
**Evolution rate analyses of** ***TaMET1*** **genes. A)** Pair-wise comparisons. Synonymous (dS), non-synonymous (dN) and evolution rate (ω) are expressed in substitution/site and were performed using *Codeml. TaMET1* from wheat homoeologous group 2 (grey boxes) were compared to *Brachypodium distachyon* [Phytozome: Bradi1g05380 and Bradi1g55287], *Sorghum bicolor* [Phytozome: Sb01g005084 and Sb02g004680], *Oriza sativa* [Genbank: AB362510 and AB362511], *Zea mays* [Phytozome: GRMZM2G333916 and GRMZM2G334041], *Hordeum vulgare* [Ensembl Genomes: MLOC_61904.6 and: MLOC_10988.2 and GenBank: CAJW010043285] and *Triticum aestivum* (*TaMET-2A1*, *2B1*, *2D1*, *5A1*, *5B1*, *5D1*, *7A1*, *7B1* and *7D1*) (accession numbers in Additional file 8). Mean values of dS, dN and ω (with ω = dN/dS) were then computed for each homoeologous group. Whiskers represent the 10-90% range of mean values, boxes represent interquartile distances, the horizontal line across whiskers represents the median, and “+” the mean values. Kruskal Wallis non-parametric tests were applied to determine significant differences between mean values (*: P < 0.05; **: P < 0.01; ***: P < 0.001). **B)** PAML branch model. Tree topology was defined by a protein alignment using the same monocot species as in A). The two monophyletic groups MET-1a and MET-1b are indicated at the right. Three distinct evolution rates ω0 (red branches), ω1 (black branches) and ω2 (blue branches) are indicated as well as the two gene duplication events described in Figure 1 (arrow).

As a first approach, pair-wise divergences were investigated between *TaMET1* genes and *MET1* genes from fully sequenced monocot species (*i.e.* divergence between orthologous pairs). Mean values for A, B and D homoeologs were then calculated per homoeologous group of chromosomes (group 2, 5 and 7) and are displayed in Figure 3A. Consistent with the neutral theory of evolution, dS rates were not significantly different between the three homoeologous groups. However significant differences were observed for dN and ω indicating a lower rate of evolution for homoeologous group 2 which belongs to the MET-1b lineage. Homoeologous group 7 is evolving at an intermediate evolution rate compared to group 2 and group 5 but does not display any deleterious mutations within the coding sequences (see also Figure 1). As expected for pseudogenes, higher dN and ω values were found for *TaMET1* at homoeologous group 5.

Secondly, various hypotheses concerning evolution rates were then tested and Likelihood Rate Tests (LRT) were computed. Eleven hypotheses were evaluated to test an increased evolution rate at various branch points in the phylogenetic tree (Additional file 1). Evolution rates are summarized in Figure 3B. The results support the existence of three evolution rates (indicated as ω0, ω1 and ω2 in Figure 3B) consistent with the two duplication events and the pair-wise analysis performed previously (Figure 3A). After gene duplication, long-term changes were observed in our phylogenetic tree. ω0, ω1 and ω2 evolution rates indicate that negative selection occurs in the MET-1b lineage, which has the smallest evolution rate (ω0 = 0.12193) suggesting its functional role in monocots. Following the first duplication event, a two fold increase in evolution rate (ω1 = 0.21988) is observed in the Met-1a lineage except for barley chromosome 5 and wheat homoeologous group 5 for which a fourfold increase (ω2 = 0.48706) is observed.

Altogether, evolution rate analyses indicate that *TaMET1* homoeologous genes of group 2 are submitted to stronger purifying selection and are evolving at a slower rate suggesting their predominant role in DNA methylation maintenance in hexaploid wheat. Following the second duplication event, asymmetric acceleration of the evolution rate is observed between homoeologous group 5 and 7 leading eventually to the formation of pseudogenes within group 5 that accumulated deleterious mutations within their coding sequences.

### Expression of *TaMET1*genes is mainly driven by homoeologous group 2

**Figure 4:**
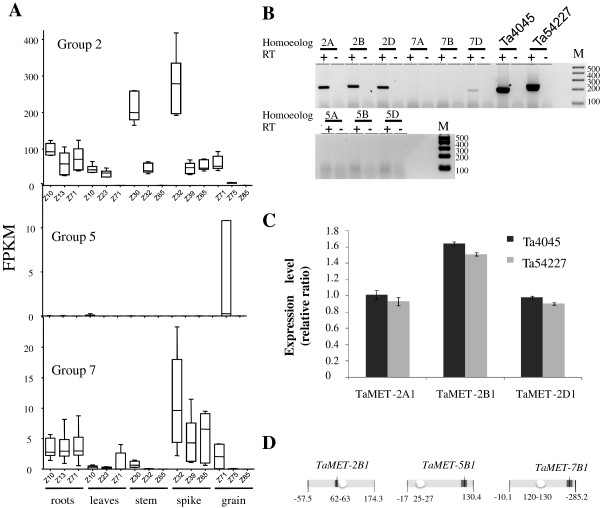
**Expression profiles of wheat** ***TaMET1*** **. A)** Wheat RNA-seq. Data are expressed in Fragment Reads per Kilobase of Exon Model (FPKM) for each homoeologous group. FPKM were computed according to the following formula: FPKM = 10^9^ x (C/NL) where C is the number of mappable reads on a feature, N is the total number of reads in the experiment and L (length) is the sum of exonic sequences in base pairs. Genes were considered to be expressed only for FPKM values >0.15. Samples covering various tissues (root, leave, stem, spike and grain) and developmental stages indicated as Zadoks (Z) scales are indicated at the bottom. Expression levels from *TaMET1* genes of a given homoeologous group were used to compute mean values. *TaMET1* from group 2 are expressed at higher levels in comparison to the other *MET1* copies and display two main peaks of expression in the stem (Z30) and spike (Z32)*.* **B)** RT-PCR gel analysis. RT-PCR with (+) or without (−) reverse transcriptase was performed for all *TaMET1* genes. Control PCR reactions were performed for two constitutively expressed genes corresponding to Ta4045 and Ta54227 selected according to Paolacci *et al.* 2009. **C)** Quantitative RT PCR. Reactions were performed for *TaMET-2A1*, *2B1* and *2D1*. Expression is relative to Ta4045 (dark grey) and Ta54227 (light grey). **D)** Schematic representation of *TaMET1* loci. Position of *MET1* genes on chromosome maps of 2B, 5B and 7A (chromosome arms in grey and centromere as white ellipse, not to scale). Top and bottom positions on ITMI reference map are indicated in cM.

The above analysis of evolution rates suggests that homoeologous genes from group 2 are under purifying selection. As it is well documented that expression patterns influence non-synonymous substitution [34], expression levels and profiles of the nine *TaMET1* genes were investigated using RNA seq data from five different organs at three developmental stages each. Expression levels of *TaMET1* homoeologous genes from group 2 were found to be 10 to 40 times higher than the *TaMET-5B1* and *TaMET* group 7 ones. For *TaMET-5A1* and *5D1*, no significant expression was detected in any of the 15 conditions (Figure 4A). Homoeologous group 2 were expressed in most tissues at nearly all developmental stages, named according to the Zadoks (Z) scale [35], but with highest expression levels at Z30 in the stem and Z32 in the spike. *MET1* expression levels in other species peak in proliferating cells such as in meristems and in reproductive organs [27, 28, 36]. In wheat we observed *TaMET1* expression at early developmental stages especially during early stem extension (Z30-Z32) when wheat is switching from the vegetative to reproductive phase. At that stage the spike tissue is proliferating requiring active replication during which DNA methylation maintenance should occur. Similarly, homoeologous group 7 were found to be expressed in almost all conditions but at a very low level compared to group 2 genes. A similar situation was observed in rice where *OsMET1a* is 10–12 times less expressed than *OsMET1b* [37]. For homoeologous group 5, only 5B is expressed at low level in grain (Figure 4A).

RNA-seq-based expression profiles were subsequently confirmed by RT-PCR. Various primer pairs were designed at the 3′UTR. Semi-quantitative and quantitative analyses confirmed the expression of *TaMET1* from homoeologous group 2 (Figures 4B and 4C) but transcripts were hardly or not detectable for group 5 and 7 (Figure 4B). Expression levels for 2A, 2B and 2D homoeologs were investigated by RT-qPCR but did not show strong differences, although 2B was found to be slightly more expressed (Figure 4C). Thus *TaMET-2A1*, *2B1* and *2D1* are expressed in an additive mode.

Recent analyses at the whole genome level indicated that housekeeping genes in wheat are enriched at pericentric positions while genes expressed with tissue-specific patterns and pseudogenes are usually found at more sub-telomeric positions [38]. To check whether there is a correlation between the observed gene expression differences and the physical position of *TaMET1* copies on the chromosomes, we genetically mapped *TaMET1* loci using 57 SNPs identified in the course of our sequence capture experiments (see Methods). Out of the 57 SNPs, 18 produced high quality results that led to the genetic mapping of five out of nine *TaMET1* genes, namely *TaMET-2B1*, *5A1*, *5B1*, *7A1* and *7B1*. As positions of homoeologous copies were consistent for groups 5 and 7, we extrapolated the position of all *TaMET1* genes from these five copies. Homoeologous group 2 were found to be located in the pericentromeric regions of the short arm of chromosomes 2 whereas group 5 and 7 were mapped to subtelomeric positions of the long arms of chromosomes 5 and 7 respectively (Figure 4D and Additional file 2).

Thus *MET1* expression is mainly driven by homoeologous group 2 indicating specific mechanisms to keep a predominant expression of homoeologous group 2 over groups 5 and 7. This observation resembles a phenomenon observed after *METI* gene duplication in Arabidopsis where *METI* transcripts accumulate to 10,000 fold higher levels than those of the duplicated *METIIa* and *b,* while *METIII* is considered to be a pseudogene [39]. Expression of a specific member of a given gene family is referred to as predominance [40] or transcriptional dominance [16] and in our case occurs for *TaMET1* genes at homoeologous group 2. The pericentric position of group 2 genes is consistent with the conclusions drawn from a recent large scale analysis indicating that genes expressed in most tissues are located in more proximal position than those displaying tissue-specific expression patterns [38]. Thus expression studies reinforce the idea that *MET1* homoeologous group 2 genes might provide methyltransferase activity.

### CG-rich isochores appear at *TaMET1*promoters and exhibit high DNA methylation

While low, or absent expression of specific *TaMET1* genes might be explained by several factors including genetic mutations or insertion of transposable elements, epigenetic marks at promoter regions are good candidates to explain differences in gene expression [23, 24, 41, 42]. Among these, cytosine methylation that occurs in CG sequence contexts has been shown to modulate gene expression in plants [23, 24, 41, 42]. To investigate the potential role of DNA methylation in the regulation of the *MET1* genes, *MET1* coding sequences as well as putative promoters were scanned for potential methylation sites in CG, CHG and CHH sequence contexts.

**Figure 5:**
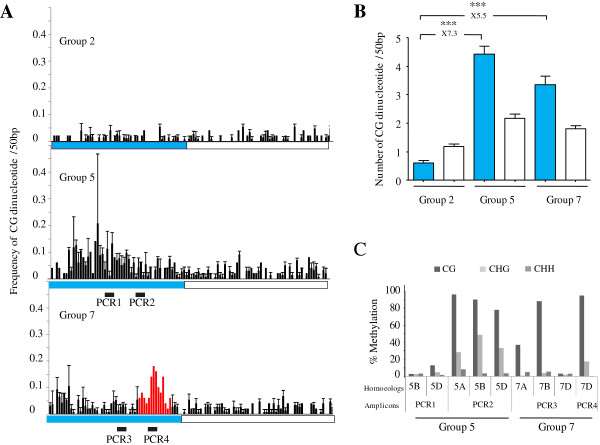
**CG enrichment and methylation at potential promoters of** ***TaMET1*** **genes from homoeologous group 5 and 7. A)** Frequency of CG dinucleotides. Frequencies were computed every 50 bp and are shown for each homoeologous group. Putative promoter region and coding sequence are delimited by respectively a blue and white box. Black bars numbered from PCR1 to PCR4 highlight the four regions studied by bisulfite sequencing and are indicated above the graphs. Region 4 is specific to the 7D homoeolog. Arrows indicate the putative transcription start site according to the RNA-seq data. **B)** Mean values of CG dinucleotides. Mean values of the number of CG dinucleotides of the three homoeologs (A, B and D) for a given homoeologous group (2, 5 and 7) in the putative promoter (blue) and coding (white) sequence regions. Differences between groups 5 and 7 putative promoter regions and group 2 are indicated above the histogram. Statistical significance was confirmed with a Kruskal Wallis non parametric tests with *: P < 0.05; **: P < 0.01; ***: P < 0.001. **C)** DNA methylation profiles as determined by bisulfite sequencing. Percentages of methylated cytosines of the four amplicons (PCR1 to 4) displayed in Figure 6A were determined after bisulfite sequencing. Percentages of methylation were recorded at each cytosine position and were used to compute a mean value for each amplicon in the CG, CHG or CHH sequence contexts.

The putative promoters of the nine genes were defined as ~2-3 kb regions upstream of the coding sequence depending upon the availability of the genomic sequences (Figure 1). Comparisons between upstream and coding sequences for potential methylation sites in CHG and CHH contexts revealed similar amounts of CHG and CHH sites for all nine genes (data not shown). In contrast, cytosines in the CG context were enriched at potential promoter regions of homoeologous group 5 (4.4 fold) and group 7 (5.5 fold) compared to group 2 putative promoters regions (Figures 5A and 5B). This result was unexpected because CG-rich regions (also known as CG-rich isochores), although already described in Arabidopsis genes, were shown to be mainly located in introns [43].

As CG-rich isochores at *TaMET1* upstream regions could be the result of new insertions of CG-rich DNA sequences, we looked for such events. Indeed, two DNA insertions of 786 and 122 bp overlapping with CG-rich isochores were observed for the *TaMET-7D1* upstream region (Additional file 3). Both insertions were already present within the ancestral D genome (*Aegilops tauschii*) suggesting their integration prior to polyploidization (Additional file 3). BLASTn analysis against the TREP database indicated a short but significant homology with a *stowaway* Miniature Inverted Repeat (MITE) for the 122 bp insertion while no significant homology was detected for the larger 786 bp insertion. BLASTn against TREP performed with the five remaining upstream regions (7A, 7B, 5A, 5B and 5D) failed to detect any transposable elements as shown in Figure 1. Instead of a new large DNA insertion enriched in CG observed at 7D, the CG-rich isochores are more dispersed along the 5A, 5B, 5D, 7A and 7B putative promoter regions (Figure 5A). This may argue in favor of a progressive CG accumulation in the course of evolution.

To determine whether these regions enriched in cytosine residues on homoeologous group 5 and 7 are indeed methylated, we performed bisulfite sequencing. We designed bisulfite primers in a way to simultaneously amplify all three homoeologous copies that we subsequently discriminated upon sequencing. Consistent with our expression studies, putative promoter regions from homoeologous groups 5 and 7 display DNA methylation in CG sequence contexts. Homoeologous group 5 also displays significant CHG methylation (Figure 5C and Additional files 4 and 5). Among all the analyzed putative promoter regions, the highest DNA methylation levels overlap with the 786 bp insertion specific to 7D (Figure 5C and Additional file 5).

Taken together, our results suggest that the presence of CG-rich isochores in the putative promoters of group 5 and 7 *TaMET1* homoeologous genes may be due to a progressive and dispersed CG-enrichment as well as to an insertion-mediated CG-enrichment, at least for the 7D copy. In addition, the high methylation levels observed in the promoter regions of the two low-expressed homoeologous groups may suggest the existence of an autoregulatory loop controlling *MET1* gene expression.

## Discussion

Bread wheat is a plant species with a large genome of about 17 Gb containing up to 80% of repetitive sequences. Much attention has been focused recently to understand how this genome, highly enriched in repetitive sequences, controls its transposable element fraction, which will otherwise lead to genome instability. One such mechanism is likely to involve DNA methylation in the CG context, which is maintained by MET1. It is therefore of importance to understand how MET1 expression is regulated in an organism with a complex hexaploid genome. In the course of our work, we observed that *TaMET1* genes contain a record of many evolutionary events, which have occurred prior and after the emergence of bread wheat.

We identified nine copies of *TaMET1* organized in three homoeologous groups at chromosomes 2, 5 and 7. At the chromosomal level, segments bearing *TaMET1* originated from two duplication events. Phylogenetic and micro-synteny confirmed that chromosome 2 and 5 paralogs originated from a WGD about 50–70 MYa in the ancestor of grass species. Then the chromosome 7 paralog emerged from a more recent gene duplication about 13–32 MYa in the *Triticea* tribe. Our analysis of the evolution rate revealed functional differences between the nine *TaMET1* genes. The MET-1b lineage (homoeologous group 2) was shown to display a lower evolution rate than the MET-1a lineage (homoeologous group 5 and 7). Lower evolution rate is observed for genes with biological function and this is best explained by purifying selection, which counter selects deleterious mutations [34, 44]. Functional significance of homoeologous group 2 genes was reinforced by our observations of expression levels and DNA methylation. Low evolution rate in the MET-1b lineage matches with a predominant expression of homoeologous group 2 over group 5 and 7. Predominant expression of one member of the *MET1* gene family was already observed in other species such as Arabidopsis [39] and rice [37] suggesting that MET1 expression level and pattern needs to be carefully controlled. Interestingly, we mapped *TaMET1* homoeologous group 2 to peri-centric (proximal) position while group 5 and 7 were located at more sub-telomeric (distal) regions. Recent large scale analyses in wheat suggested that distal regions are more dynamic, displaying higher level of recombination and accumulate more pseudogenes and gene duplications than proximal peri-centric regions [38]. Furthermore, genes at distal position display more tissue specific expression than those at more proximal position. It is then tempting to hypothesise that a distal chromosome position may have a direct influence on expression leading as a consequence to the predominance of the more proximal genes as observed in our case for homoeologous group 2. Homoeologous group 2 did not show any differences in gene expression among the three homoeologs. Consistent with the whole genome analyses was the fact that genome-wide transcriptional dominance of an individual subgenome (A, B or D) was not observed [18]. Besides its position along the chromosome, our data indicated that DNA methylation observed in the promoter region of homoeologous group 5 and 7 may have contributed to their transcriptional repression and may have favored an increased evolution rate at *TaMET-5A1* and *5D1* leading to the accumulation of deleterious mutations, a process known as pseudogenization [45, 46]. Interestingly, distinctions can be made between group 5 homoeologs: 5A already accumulated large deletions and numerous stop codons before polyploidization, while stop codons occurred at 5D after polyploidization but are absent at 5B which however displays an elevated level of non-synonymous mutations and is expressed only in grains. Pseudogenes are usually rapidly eliminated and the fact that *TaMET-5A1* and *5D1* pseudogenes are maintained suggests that pseudogenization may not be fully achieved or that these genes contribute in a significant but yet unknown manner to TaMET1 activity.

Our data support a functional role of DNA methylation in the initiation or the maintenance of gene silencing at specific *TaMET1* genes. Considering that the chromosome 2 paralog is the ancestral locus and shows low occurrence of potential CG methylation sites, the observed CG-rich isochores at chromosome 5 and 7 paralogs associated with DNA methylation imply CG-enrichment at these putative promoter regions. CG-enrichment was unexpected as usually CG dinucleotides are under-represented due to 5-methylcytosine deamination [43]. At the moment we can only speculate about their possible origin. First, GC-rich and GC-poor isochores are known to occur in animals and several hypotheses have been proposed to explain their emergence [47]. Among them the GC-biaised gene conversion (gBGC) has been proposed as one of the main driving forces in the evolution of nucleotide composition. gBGC favors GC over AT bases in alleles during mismatch repair following heteroduplex formation in the course of meiosis. gBGC results from Base Excision Repair (BER) and involves a DNA glycosylase that specifically removes thymine in DNA heteroduplexes. Secondly, animal genomes display unmethylated CG-rich elements known as CpG islands (CGIs). CGIs are defined as DNA sequences of a few hundred base pairs, with high CG occurrence, high G + C frequency and are involved in the regulation of gene expression [48]. CGIs have been divided into start and non-start CGIs. Non-start CGI are the most abundant and best explained by insertion of repeated sequences such as transposable elements (in the human genome 79% are due to *Alus*) while start CGIs located at the transcription start sites are only poorly explained by transposable element insertion (in the human genome 5,6% are due to *Alus*) [49]. Interestingly, Suzuki et al., [50] also proposed gBGC as one of the possible mechanisms to explain the emergence of start CGIs. Recently, it was suggested that gBGC occurs in plants [51]. gBGC can be considered as one of the possible mechanisms explaining the emergence of CG-rich isochores at *TaMET1* putative promoter regions. Indeed, it may be an attractive mechanism to explain the progressive CG enrichment we observed at *TaMET1* upstream regions especially at homoeologous group 5 and 7 located at distal chromosome positions where higher recombination rates have been reported [38, 52]. Furthermore, the *MET-7D1* copy would have also undergone insertion of CG-rich DNA fragments in a mechanism very reminiscent to the one observed for non-start CGIs, arguing for shared evolutionary mechanisms between animal and plants toward the emergence of CG-rich isochores.

Once CG-rich isochores appeared, they can be methylated in order to silence gene expression. Although CGIs were not described in plant promoters, “dense CG methylation clusters” have been reported and are proposed to silence cryptic promoters within the coding sequence [43]. Silencing of these cryptic promoters is established first through the RNA-directed DNA Methylation (RdDM) pathway and results in methylation at cytosine residues at CG, CHG and CHH sequence contexts. Once methylation is set up, only methylation in the CG context, which does not rely on siRNA production, can be maintained in the course of evolution leading to high methylation only in CG sequence contexts [43]. If such a mechanism occurred within the putative promoter region of *TaMET1* genes, it can explain how homoeologous group 7 became progressively repressed.

Given the correlation between DNA methylation in promoter regions and gene silencing [23, 24], we suggest that DNA methylation may be part of a possible auto-regulatory mechanism among *TaMET1* genes. In this model, MET1 mainly encoded by homoeologous group 2 regulates group 7 gene expression through CG DNA methylation maintenance. CG methylation at homoeologous group 7 may be alleviated in specific organs, developmental stages or upon changing environmental conditions. However possible roles for the homoeologous group 7 (MET1-a lineage) is challenged by recent data collected in rice indicating that the main MET1 function is ensured by *Met1b* and not *Met1a*. Indeed, RNAi against *Met1a* does not significantly affect plant development while a *met1b* null mutant is lethal [28, 53].

## Conclusions

**Figure 6:**
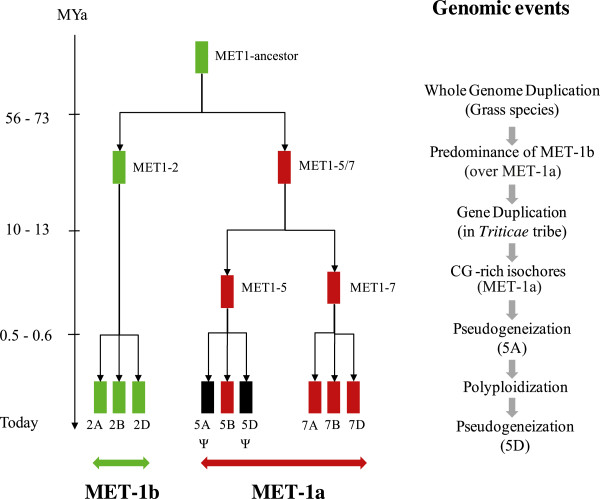
**Scheme of the genome dynamics at** ***TaMET1*** **loci in the course of evolution.** Scheme of the emergence of the nine *TaMET1* genes is given at the left. Evolution time is given from the top to the bottom in MYa. The MET-1a lineage is expressed (green) and evolved at a low evolution rate while genes in the MET-1b lineage are repressed (red) or evolved as pseudogenes (Ψ, black boxes). The hypothetic succession of genomic events occurring at or including *TaMET1* is proposed at the right.

From our data, we propose a chronology (Figure 6) of the genomic events observed at *TaMET1* genes, which include WGD, gene duplication, expression predominance of homoeologous group 2, CG-rich isochores emergence, DNA methylation and pseudogenization. The unexpectedly rich evolution history observed at *TaMET1* makes these loci a very attractive model to study further gene evolutionary mechanisms occurring in hexaploid wheat. Increased copy number finally leads to *TaMET1* silencing at homoeologous group 5 and 7 (the MET-1a lineage), keeping genes of group 2 (the MET-1b lineage) in an active state. We hypothesize that CG methylation was used as a mean to control gene expression in the MET-1a lineage favoring low expression at homoeologous group 7 and pseudogenization at group 5. For the latter the different evolutionary stages are still observed between homoeologs. CG methylation might be required to limit homoeologous group 7 transcription using CG-rich isochores, which are the target of CG DNA methylation. At that point, we can only speculate about the possible involvement of methylation in limiting homoeologous group 7 expression in tissues or developmental stages where group 2 is expressed, maybe leading to tissue-specific expression patterns of group 7 genes and their subfunctionalization.

Taken together, our data suggest that DNA methylation at *TaMET1* loci can act as an epigenetic determinant required to drive genome evolution.

## Methods

### Analysis of IWGSC sequence surveys

Access to the IWGSC physical map sequences from hexaploid wheat *cv Chinese Spring* has been established by the URGI (http://urgi.versailles.inra.fr/) on the behalf of IWGSC. BLASTn analyses were performed at the URGI database. Identification of transposable elements in selected IWGSC contigs was performed by BLASTn analyses against the TREP database at http://wheat.pw.usda.gov/ITMI/Repeats/. CG, CHG and CHH profiles (where H is A, C or T) were detected using an in house Perl script available upon request. Gene structures were predicted by cDNA alignment against genomic sequences using *SIM4* (http://pbil.univ-lyon1.fr); protein domains are according to the Pfam database (http://pfam.sanger.ac.uk/). Gene structures were subsequently designed by *FancyGene* (http://bio.ieo.eu/fancygene/).

### Sequence capture

Sequence capture experiments [54, 55] were designed to isolate and sequence DNA segments using probes synthesized on a microarray. Two runs of sequence capture were performed according to the NimbleGen Arrays User’s Guide followed by 454 Optimized Sequence Capture method. Briefly, *MET1* specific probes ranging from 60 to 90 nucleotides were designed at a density of ~1.10^6^ probes/Mb of sequence by Roche-Nimblegen from one *Triticum aestivum* public EST (accession number TA8302), one private EST (GPIC:S:720428) and from the two coding sequences from *Brachypodium distachyon* (Accession numbers [Phytozome: Bradi1g05380 and Bradi1g55290]). The absence of repeated sequences was verified using repeatMasker and TREP release 10 database. Genomic DNA from wheat elite lines cv *Brigadie*r, *Alcedo*, *Renan* and *Recital* were used to build up four distinct genomic DNA libraries by nebulization with an average of fragment sizes of ~600 bp. Libraries were then hybridized onto capture arrays, captured DNA was eluted and amplified prior to 454 sequencing on a GS FLX Titanium platform according to the manufacturer. Overall, sequence captures yielded 8,184 reads specific to *MET1* and *de novo* assembly was subsequently performed using *gsAssembler* (Roche) with specific parameters set at 98% similarity and 20 bp overlap. Sequences were verified in the course of the project by BLASTn analysis against the IWGSC surveys and by PCR amplification on diploid and hexaploid species.

### Genetic mapping

Two mapping populations were used: recombinant Inbred Lines derived from a cross between *Triticum aestivum cv Renan* and *Recital* [56] and a doubled haploid population derived from a cross between *Triticum aestivum cv Brigadier* and *Alcedo* (Biogemma personal communication). DNA from all four elite lines was used in the sequence capture experiments and reads were grouped according to the four DNA origins. As a whole, 57 putative Single Nucleotide Polymorphisms (SNPs) were identified and genotyping was subsequently performed on genomic DNA from the two mapping populations using KASPar (KBioscience) fluorescent competitive allele-specific amplification. Primers were designed with *Primer picker* (KBioscience) and PCR amplifications were performed on a hydrocycler (LGC genomics), for 41 to 50 cycles at 57°C and then run onto a Genotyper (Applied Biosystem). The list of primers used to perform the genetic mapping is provided in Additional file 6. SNP mapping was performed on the two genetic maps using an in-house bioinformatic pipeline available at Biogemma. Genetic positions are given according to the *Renan* x *Recital* recombination map [56]. Physical positions are according to the names of the IWGSC contigs obtained by BLASTn analysis against the virtual map and are included within the virtual map designed by synteny.

### Phylogenetic reconstruction and substitution rate calculation

*TaMET1* coding sequences were used for phylogenetic reconstruction and substitution rate calculation. Selected sequences were first aligned with *MUSCLE* multiple sequence alignment [57] and then refined using *Gblocks* [58]. Maximum likelihood analysis was performed with *PhyML* using default parameters with 1,000 bootstraps [59]. Phylogenetic trees were drawn using *ITOL* [60]. Substitution rate studies were performed as follows: first, a new phylogenetic tree was built with the same species except that here, dicot species were not considered and the tree was based on protein sequences instead of cDNA. For *TaMET1*, genomic sequences were used to predict exonic sequences using *FGENESH* [61] and subsequently assembled into a predicted cDNA. Predicted cDNAs were validated in the course of this study by RNA-seq data. cDNAs were translated using *Transeq* and *Sixpack* from the *EMBOSS* package [62]. The phylogenetic tree was then built from predicted proteins as described above. ω (the ratio of nonsynonymous/synonymous substitution rates) was determined using *Codeml* from the *PaML* package [33]. A likelihood ratio test (LRT) was used to compare various hypothesis models in which ω values are expected to differ among branches, in comparison to a null hypothesis in which all the branches have similar ω. LRT values were then compared to a chi-squared distribution with degrees of freedom equal for a given tree to the number of values of ω -1, as described in Yang [33]. The phylogenetic data sets supporting the results of this article are available in the TreeBASE repository [http://purl.org/phylo/treebase/phylows/study/TB2:S16421]. The data supporting the evolution rate investigated in this study are included within the article and its additional files (Additional file 1).

### Micro-synteny analyses

Starting from Murat *et al.* [31], chromosomal segments including *MET1* loci were selected from rice and brachypodium. To be able to compare our results with those of Murat *et al.* [31], the same fragment boundaries were retained but in our case, all the coding sequences of a given genomic fragment have been considered. Briefly, rice chromosome 3 [Phytozome: LOC_Os03g58040.1 to LOC_Os03g58920.1] (covering 510.70 kb of genomic DNA and including 80 genes) and brachypodium chromosome 1 [Phytozome: Bradi1g05680 to Bradi1g04980] (covering 531.9 kb of genomic DNA and including 72 genes) chromosomal segments are syntenic to wheat chromosome 5 and 7 (Additional file 7). Gene sequences from model species were then used to perform BLASTn analysis against the IWGSC sequence surveys as described in Salse et al. [11] using 70% CIP (Cumulative Identity Percentage) but only 30% CALP (Cumulative Alignment Length Percentage). The CALP parameter was kept at a low value in order to detect all the micro-syntenic relationships. Percentage of syntenic conservation was then computed as 100 × the number of conserved genes in wheat/number of genes in the syntenic region from brachypodium or from rice. The data set supporting the results is included within the article and its additional files (Additional file 7).

### RNA-seq

RNA-seq non-oriented libraries were constructed in two replicates from total RNAs of hexaploid wheat *cv Chinese Spring*. RNAs were prepared with the TruSeq kit (Illumina) for 15 biological samples including 5 organs (root, leaves, stem, spike, grain) and 3 developmental stages (beginning, middle, and end of development) as described in [63] (Additional file 8). For oriented libraries, samples were pooled by organs, rRNAs were removed from total RNAs with the riboZero kit (Ambion) and RNA-seq libraries were constructed with the ScriptSeq kit (Epicentre). All the libraries were sequenced using a HiSeq200 (Illumina) with reads of 100 bp sequenced in both directions. Reads from RNA-seq libraries were mapped using *Tophat2 v2.0.8* [64] and *Bowtie2* [65] onto the *MET1* genomic sequences with neither mismatches nor splice-mismatches allowed. Transcript reconstruction and expression levels were analyzed with *Cufflinks v2.0.2* [66] without annotation. Because sequencing was bidirectional, which is to say that two reads correspond to the same cDNA molecule, expression data results of transcription levels are expressed in Fragments per Kilobase of Exon Model (FPKM) per million mapped reads [67]. The RNA-seq data sets supporting the results of this article are available in the Sequence Read Archive (SRA) repository, [http://www.ncbi.nlm.nih.gov/sra/ERP004714].

### RNA analyses

Wheat plantlets of *cv Chinese Spring* were grown in a greenhouse and collected at Z61-65 stage according to Zadoks scale [35]. Tissues were frozen in liquid nitrogen and ground to a fine powder. Total RNAs were extracted from 250 mg of plant material using an RNA extraction method adapted from [68]. RNA was subsequently treated with 100 units of DNase I (Invitrogen) in the presence of 20U RNaseOUT™ Recombinant Ribonuclease Inhibitor (Invitrogen). Quantity of extracted RNA was estimated using a Nanodrop (Thermo Scientific) and RNA quality was checked by migration on a 2% agarose gel containing MOPS 2% and 0,05% formaldehyde.

Reverse Transcription was performed from 2 μg of total RNA using an oligo(dT) 15 Primer and M-MLV Reverse Transcriptase (Promega) in presence of Recombinant RNasin Ribonuclease Inhibitor (Promega) according to the supplier’s recommendation. Homoeologous specific primers were designed manually and validated with *Oligo Analyzer* (Gene Link) to avoid secondary structure formation. Sequences of selected primer pairs can be found in Additional file 6. Semi-quantitative analyses were performed using primer pairs with similar efficiencies and on the same cDNA sample by comparing the *TaMET1* PCR product to Ta4045 and Ta54227 as reference genes (primer pairs as as in [69]). Quantitative analysis was performed on a LightCycler® 480 System using LightCycler® 480 SYBR Green I Master reagent (Roche) according to the supplier’s recommendation. Primer pair efficiencies were calculated through serial dilutions from 1/3 to 1/81 for each RNA sample and only primer pairs with a PCR efficiency between 80 and 110% were selected. As in semi-quantitative analyses, Ta4045 and Ta54227 were used as reference genes.

### Bisulfite sequencing

1 g of plant material was collected from stem and leaves at the Z30 stage and DNA extracted using the DNeasy plant maxi kit (Qiagen). 200-500 ng of DNA was subjected to bisulfite (BS) treatment using the EZ DNA Methylation-Gold™ Kit (Zymo Research). BS-treated DNA was PCR-amplified using specific primers (Additional file 6) and cloned in pGEMT vectors (Promega) prior to sequencing. 10–20 clones were analyzed for each genomic region using Kismeth software [70]. Two PCR fragments from the VERNALIZATION1 (*VRN1*) gene previously studied by bisulfite experiments [71] were used as internal controls. Incomplete conversion was excluded by analyzing the 0.0 k fragment from *VRN-A1,* which is devoid of CG methylation, while optimal bisulfite treatment were assessed by analysis of the 9.2 k fragment, a highly CG methylated region from *VRN-A1*. Examples of results are given in Additional file 9.

### Availability of supporting data

The following additional data is available with the online version of this paper. Additional file 1 is a table listing the results of the Likelihood ratio tests. Additional file 2 is a table listing the genetic positions of *TaMET1* loci. Additional file 3 is a sequence alignment of the promoter region of *TaMET1* from homoeologous group 7 with close species. Additional files 4 and 5 are detailed bisulfite analyses performed at *TaMET1* from homoeologous group 5 and 7 respectively. Additional file 6 is a table listing the primers used in this study. Additional file 7 is a table describing micro-synteny data between wheat, rice and brachypodium. Additional file 8 is a table listing the RNA-seq samples used in this study. Additional file 9 is an example of control experiment in bisulfite sequencing analysis.

## Electronic supplementary material

Additional file 1: **Likelyhood ratio tests (LRT). A)** Likelihood ratio test (LRT). LRT has been used to compare 11 hypotheses (H_1–11_) in respect to the null hypothesis (H_0_) in which all the branches have the same evolution rate (ω0). Hypotheses were designed to test if the MET1 phylogenetic tree includes up to three evolution rates (ω0, ω1 and ω2) and if these variations in ω values are long term changes (*i.e.* if all the branches below a duplication event display the same ω value) or increase only after a duplication event and then is relaxed to ω0. **B)** Details of the 11 hypotheses tested in the branch model described in Figure 3B. For each hypothesis tested, a tree file in Newick format and a graphic representation highlighting the branches considered in the tested hypothesis are given. (PDF 472 KB)

Additional file 2: **Genetic positions of** ***TaMET1*** **loci.** Distal and proximal markers from the ITMI reference map and flanking the 2B, 5B and 7A *TaMET1* loci are given in cM. (PDF 9 KB)

Additional file 3: **Alignment at putative promoter regions of** ***TaMET1*** **genes from homoeologous group 7.** *Hordeum vulgare* chromosome 7 [Ensembl Genomes: MLOC_10988.2], *Triticum aestivum* chromosome 7A [IWGSC: 7AL:4532056], 7B [IWGSC: 7BL:6682174] and 7D [IWGSC: 7DL:3392185], *Triticum urartu* chromosome 7 [Ensembl Genomes: scaffold38640], *Triticum tauschii* chromosome 7 [Ensembl Genomes: scaffold2203], Alignment were performed with *MUSCLE* and refined by *jalview*. (PDF 501 KB)

Additional file 4: **Bisulfite analysis of putative promoter region of homoeologous group 5. A)** Frequencies of CG dinucleotides were computed every 50 bp of the putative promoter regions of homoeologous group 5. 5A (black), 5B (white) and 5D (grey). Black bars numbered from 1 to 4 highlight the two regions studied by bisulfite sequencing. **B)** Kismeth outputs of the percentage of methylated cytosines in CG (red), CHG green) and CHH (blue) context. (PDF 172 KB)

Additional file 5: **Bisulfite analysis of putative promoter region of homoeologous group 7.** **A)** Frequencies of CG dinucleotides were computed every 50 bp of the putative promoter regions of homoeologous group 7. 7A (black), 7B (white) and 7D (grey). Black bars numbered from 1 to 4 highlight the two regions studied by bisulfite sequencing. **B)** Kismeth outputs of the percentage of methylated cytosines in CG (red), CHG green) and CHH (blue) context. (PDF 155 KB)

Additional file 6: **Primers used in RT-PCR, RT-qPCR, mapping and bisulfite experiments.** (PDF 20 KB)

Additional file 7: **Virtual physical map reconstruction at** ***TaMET1*** **loci from micro-synteny data.** Physical maps for Os and Bd, virtual physical map based on IWGSC surveys organized from rice and brachypodium orthologs. *TaMET1* loci are highlighted in yellow. Note that two overlapping contigs were found at *TaMET-5A1* indicating that these two IWGSC contigs were not assembled together in the course of the assembly process. (PDF 21 KB)

Additional file 8: **RNA-seq samples used in this study.** (PDF 33 KB)

Additional file 9: **Controls in bisulfite experiments. A)** Methylation rates at two VRN-A1 regions called 0.0 k and 9.2 k (adapted from [71]). **B)** Structure of the *VRN-A1* gene. **C)** Typical results from bisulfite experiments for 0.0 k (no CG methylation) and 9.2 k (high CG methylation). (PDF 2 MB)

## Abbreviations

BAH: Bromo-adjacent homology
BER: Base excision repair
CALP: Cumulative alignment length percentage
CIP: Cumulative identity percentage
CGIs: CpG islands
DNMT1-RFD: Cytosine specific DNA methyltransferase replication foci domain
LRT: Likelihood rate tests
ω: Evolution rate
ESTs: Expressed sequence tags
FPKM: Fragments per kilobase of exon model
gBGC: GC-biaised gene conversion
IWGSC: International wheat genome sequencing consortium
MET1: METHYLTRANSFERASE1
MYa: Million years ago
MITE: Miniature inverted repeat
dN: Rate of non-synonymous substitution
dS: Rate of synonymous substitution
RdDM: RNA-directed DNA Methylation
SNP: Single nucleotide polymorphism
WGD: Whole-genome duplication
Z: Zadoks scale.
