# Supplementary material for: Evolutionary history of Methyltransferase 1 genes in hexaploid wheat
Source: BMC Genomics. 2014 Oct 23;15(1):922. doi: 10.1186/1471-2164-15-922 (PMC4223845; doi:10.1186/1471-2164-15-922)

Additional file 1

Additional file 1A: Likelihood ratio tests (LRT)

Hypothesis	*Lnl*	*2Δl*	*ddl*	LRT	ω0	ω1	ω2	P<0.05	P<0.01
h0	-21434.8	-	-	-	0.23993	-	-	-	-
h1	-21330.6	104.1	1	0	0.17544	0.55407	-	***	***
h2	-21434.3	0.4768	1	0.0383	0.23822	0.30632	-	*	ns
h3	-21434.0	0.7571	1	0.0383	0.23567	0.26794	-	*	ns
h4	-21434.8	0.0009	1	1	0.23986	0.24225	-	ns	ns
h5	-21362.2	72.6	1	0	0.12974	0.32857	-	***	***
h6	-21434.8	-0.00004	1	1	0.23993	0.36697	-	ns	ns
h7	-21362.2	72.6	1	0	0.12974	0.32858	-	***	***
h8	-21434.6	0.2523	1	0.0617	0.21638	0.2417	-	ns	ns
h9	-21433.4	1.4	1	0.0235	0.22161	0.2512	-	*	ns
h10	-21330.6	104.1	1	0	0.17544	0.55407	-	***	***
h11	-21326.0	108.8	2	0	0.12193	0.21988	0.48706	***	***

Additional file 1B: Details of the 11 hypotheses tested in the branch model described in Fig 3B

h0: all the branches evolve at the same evolution rate
(((((TaMet-2D1,(TaMet-2A1,(TaMet-2B1,HvMet1-2))),BdMet1b),OsMet1b),((ZmMet1b-2,SbMet1b),ZmMet1b-1)),((((HvMet1-5,(TaMet-5A1,(TaMet-5D1,TaMet-5B1))),((HvMet1-7,TaMet-7B1),(TaMet-7A1,TaMet-7D1))),BdMet1a),(OsMet1a,SbMet1a)));

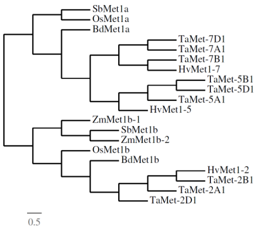

h1: the branches including group 5 homoeologs and HvMet1-5 evolve at the same rate which is significantly different from the rest of the tree
(((((TaMet-2D1,(TaMet-2A1,(TaMet-2B1,HvMet1-2))),BdMet1b),OsMet1b),((ZmMet1b-2,SbMet1b),ZmMet1b-1)),((((HvMet1-5 #1,(TaMet-5A1 #1,(TaMet-5D1 #1,TaMet-5B1 #1) #1) #1) #1,((HvMet1-7,TaMet-7B1),(TaMet-7A1,TaMet-7D1))),BdMet1a),(OsMet1a,SbMet1a)));

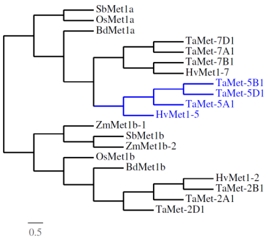

h2: evolution rate increases soon after the second duplication and return to its initial rate
(((((TaMet-2D1,(TaMet-2A1,(TaMet-2B1,HvMet1-2))),BdMet1b),OsMet1b),((ZmMet1b-2,SbMet1b),ZmMet1b-1)),((((HvMet1-5,(TaMet-5A1,(TaMet-5D1,TaMet-5B1)))#1, ((HvMet1-7,TaMet-7B1),(TaMet-7A1,TaMet-7D1))),BdMet1a),(OsMet1a,SbMet1a)));

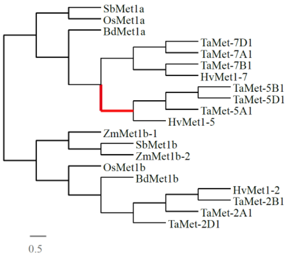

h3: the branches including group 7 homoeologs and HvMet1-7 evolve at the same rate which is significantly different from the rest of the tree
(((((TaMet-2D1,(TaMet-2A1,(TaMet-2B1,HvMet1-2))),BdMet1b),OsMet1b),((ZmMet1b-2,SbMet1b),ZmMet1b-1)),((((HvMet1-5,(TaMet-5A1,(TaMet-5D1,TaMet-5B1 #1))),((HvMet1-7 #1,TaMet-7B1 #1) #1,(TaMet-7A1 #1,TaMet-7D1 #1) #1) #1),BdMet1a),(OsMet1a,SbMet1a)));

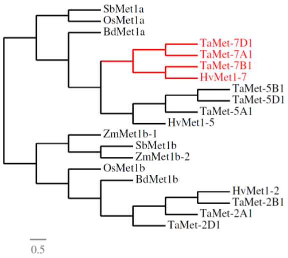

h4: evolution rate increases soon after the second duplication and return to its initial rate

(((((TaMet-2D1,(TaMet-2A1,(TaMet-2B1,HvMet1-2))),BdMet1b),OsMet1b),((ZmMet1b-2,SbMet1b),ZmMet1b-1)),((((HvMet1-5,(TaMet-5A1,(TaMet-5D1,TaMet-5B1))),((HvMet1-7,TaMet-7B1),(TaMet-7A1,TaMet-7D1)) #1),BdMet1a),(OsMet1a,SbMet1a)));

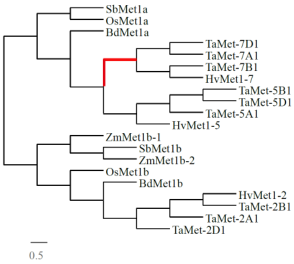

h5: the branches including MET1a lineage evolve at the same rate which is significantly different from the rest of the tree (i.e from MET1b lineage)
(((((TaMet-2D1,(TaMet-2A1,(TaMet-2B1,HvMet1-2))),BdMet1b),OsMet1b),((ZmMet1b-2,SbMet1b),ZmMet1b-1)),((((HvMet1-5 #1,(TaMet-5A1 #1,(TaMet-5D1 #1,TaMet-5B1 #1) #1) #1) #1,((HvMet1-7 #1,TaMet-7B1 #1) #1,(TaMet-7A1 #1,TaMet-7D1 #1) #1) #1) #1,BdMet1a #1) #1,(OsMet1a #1,SbMet1a #1)#1) #1);

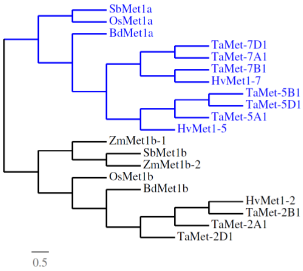

h6: evolution rate increases soon after the first duplication and return to its initial rate
(((((TaMet-2D1,(TaMet-2A1,(TaMet-2B1,HvMet1-2))),BdMet1b),OsMet1b),((ZmMet1b-2,SbMet1b),ZmMet1b-1)),((((HvMet1-5,(TaMet-5A1,(TaMet-5D1,TaMet-5B1))),((HvMet1-7,TaMet-7B1),(TaMet-7A1,TaMet-7D1))),BdMet1a),(OsMet1a,SbMet1a)) #1);

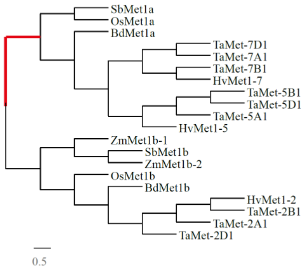

h7: the branches including MET1b lineage evolve at the same rate which is significantly different from the rest of the tree (i.e from MET1b lineage)
(((((TaMet-2D1 #1,(TaMet-2A1 #1,(TaMet-2B1 #1,HvMet1-2 #1) #1) #1) #1,BdMet1b #1) #1,OsMet1b#1) #1,((ZmMet1b-2 #1,SbMet1b #1) #1,ZmMet1b-1 #1) #1) #1,((((HvMet1-5,(TaMet-5A1,(TaMet-5D1,TaMet-5B1))),((HvMet1-7,TaMet-7B1),(TaMet-7A1,TaMet-7D1))),BdMet1a),(OsMet1a,SbMet1a)));

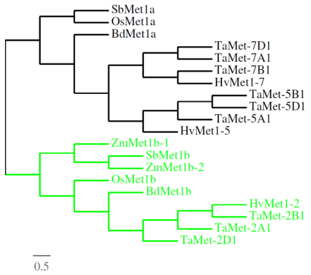

h8: evolution rate increases soon after the first duplication and return to its initial rate

(((((TaMet-2D1,(TaMet-2A1,(TaMet-2B1,HvMet1-2))),BdMet1b),OsMet1b),((ZmMet1b-2,SbMet1b),ZmMet1b-1)) #1,((((HvMet1-5,(TaMet-5A1,(TaMet-5D1,TaMet-5B1))),((HvMet1-7,TaMet-7B1),(TaMet-7A1,TaMet-7D1))),BdMet1a),(OsMet1a,SbMet1a)));

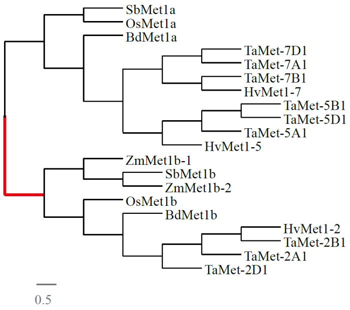

h9: the branches including MET1a lineage except group 5 homoeologs and HvMet1-5 evolve at the same rate which is significantly different from the rest of the tree
(((((TaMet-2D1,(TaMet-2A1,(TaMet-2B1,HvMet1-2))),BdMet1b),OsMet1b),((ZmMet1b-2,SbMet1b),ZmMet1b-1)),((((HvMet1-5,(TaMet-5A1,(TaMet-5D1,TaMet-5B1))),((HvMet1-7 #1,TaMet-7B1 #1) #1,(TaMet-7A1 #1,TaMet-7D1 #1) #1) #1) ,BdMet1a #1) #1,(OsMet1a #1,SbMet1a #1)#1) #1);

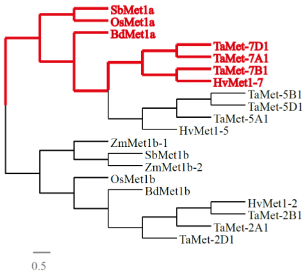

h10: all branches except group 5 homoeologs and HvMet1-5 evolve at a different rate which is significantly different from group 5 homoeologs and HvMet1-5 (reciprocal to h1)

(((((TaMet-2D1 #1,(TaMet-2A1 #1,(TaMet-2B1 #1,HvMet1-2 #1) #1) #1) #1,BdMet1b #1) #1,OsMet1b#1) #1,((ZmMet1b-2 #1,SbMet1b #1) #1,ZmMet1b-1 #1) #1) #1,((((HvMet1-5,(TaMet-5A1,(TaMet-5D1,TaMet-5B1))),((HvMet1-7 #1,TaMet-7B1 #1) #1,(TaMet-7A1 #1,TaMet-7D1 #1) #1) #1) #1,BdMet1a #1) #1,(OsMet1a #1,SbMet1a #1) #1) #1);

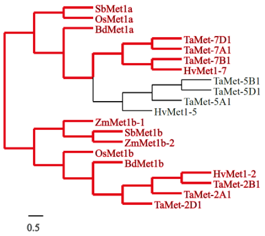

h11: 3 evolution rates are observed, one for branches including group 5 homoeologs and HvMet1-5, one for group 7 homoeologs and HvMet1-7 and one for the MET1b lineage

(((((TaMet-2D1 #2,(TaMet-2A1 #2,(TaMet-2B1 #2,HvMet1-2 #2) #2) #2) #2,BdMet1b #2),OsMet1b #2) #2,((ZmMet1b-2 #2,SbMet1b #2) #2,ZmMet1b-1 #2) #2) #2,((((HvMet1-5,(TaMet-5A1,(TaMet-5D1,TaMet-5B1))),((HvMet1-7 #1,TaMet-7B1 #1) #1,(TaMet-7A1 #1,TaMet-7D1 #1) #1) #1) ,BdMet1a #1) #1,(OsMet1a #1,SbMet1a #1)#1) #1);

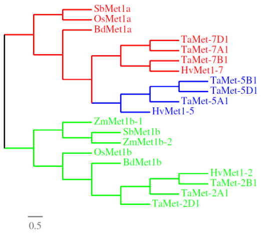

Supplement: Supplementary file 1 — Additional file 1: Likelyhood ratio tests (LRT). A) Likelihood ratio test (LRT). LRT has been used to compare 11 hypotheses (H1–11) in respect to the null hypothesis (H0) in which all the branches have the same evolution rate (ω0). Hypotheses were designed to test if the MET1 phylogenetic tree includes up to three evolution rates (ω0, ω1 and ω2) and if these variations in ω values are long term changes (i.e. if all the branches below a duplication event display the same ω value) or increase only after a duplication event and then is relaxed to ω0. B) Details of the 11 hypotheses tested in the branch model described in Figure 3B. For each hypothesis tested, a tree file in Newick format and a graphic representation highlighting the branches considered in the tested hypothesis are given. (PDF 472 KB) [file 12864_2014_6631_MOESM1_ESM.pdf]
