# Supplementary material for: Evolutionary history of Methyltransferase 1 genes in hexaploid wheat
Source: BMC Genomics. 2014 Oct 23;15(1):922. doi: 10.1186/1471-2164-15-922 (PMC4223845; doi:10.1186/1471-2164-15-922)
Supplement: Supplementary file 2 — Additional file 2: Genetic positions of TaMET1 loci. Distal and proximal markers from the ITMI reference map and flanking the 2B, 5B and 7A TaMET1 loci are given in cM. (PDF 9 KB) [file 12864_2014_6631_MOESM2_ESM.pdf]

Additional file 2

	2B		5B		7A	
Position	ITMI	Position/top	ITMI	Position/top	ITMI	Position/top
proximal	Xgpw4382-2B	60.4	Xgwm604-5B	101.5	Xcfa2040-7A	226.5
distal	Xgpw2225-2B	60	Xfbb323-5B	121.2	Xgpw4050-7A	275.8
