# Supplementary figures and images for: Evolutionary history of Methyltransferase 1 genes in hexaploid wheat
Source: BMC Genomics. 2014 Oct 23;15(1):922. doi: 10.1186/1471-2164-15-922 (PMC4223845; doi:10.1186/1471-2164-15-922)

Additional file 3

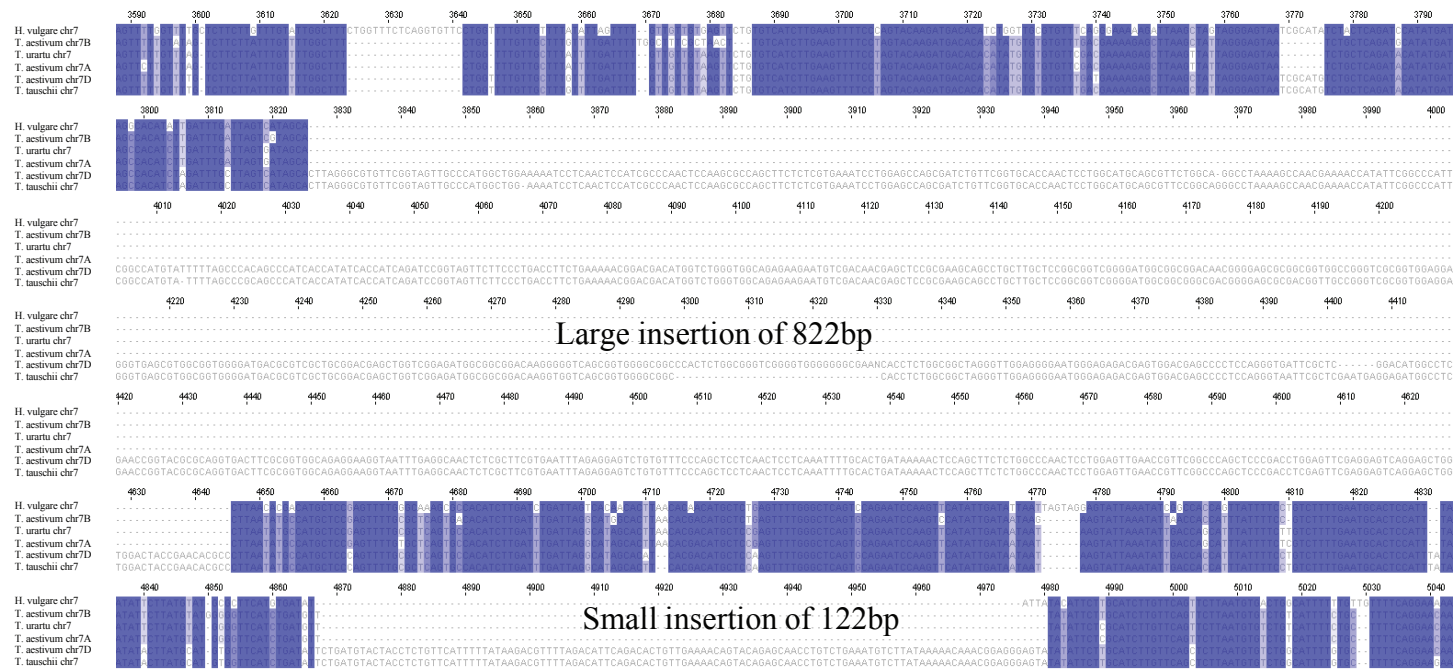

Supplement: Supplementary file 3 — Additional file 3: Alignment at putative promoter regions of TaMET1 genes from homoeologous group 7. Hordeum vulgare chromosome 7 [Ensembl Genomes: MLOC_10988.2], Triticum aestivum chromosome 7A [IWGSC: 7AL:4532056], 7B [IWGSC: 7BL:6682174] and 7D [IWGSC: 7DL:3392185], Triticum urartu chromosome 7 [Ensembl Genomes: scaffold38640], Triticum tauschii chromosome 7 [Ensembl Genomes: scaffold2203], Alignment were performed with MUSCLE and refined by jalview. (PDF 501 KB) [file 12864_2014_6631_MOESM3_ESM.pdf]

Additional file 4

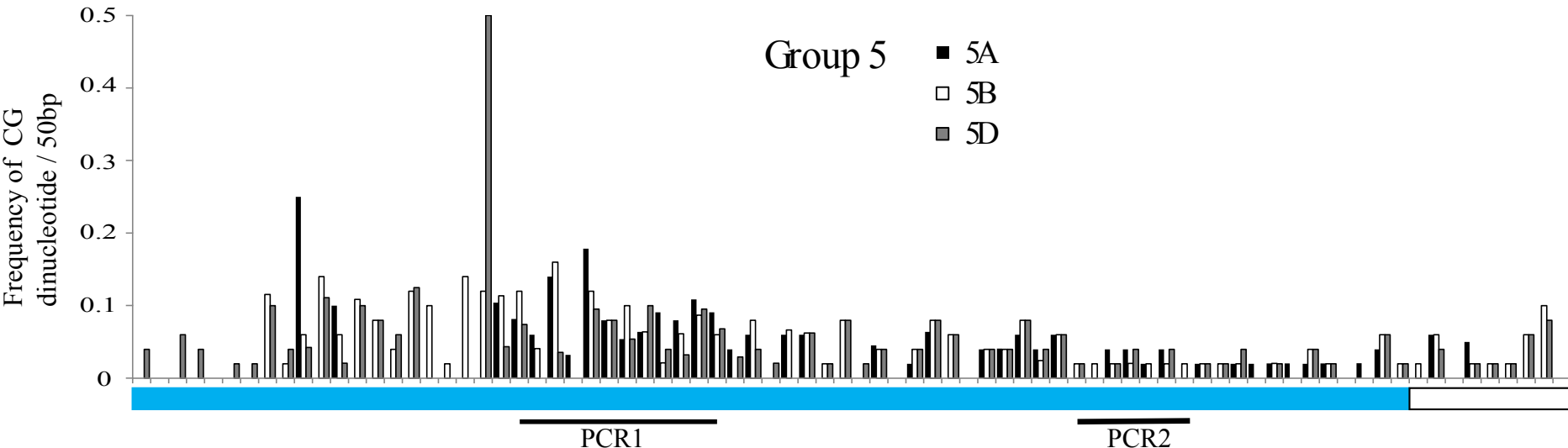

- CG
- CHG
- CHH

5A

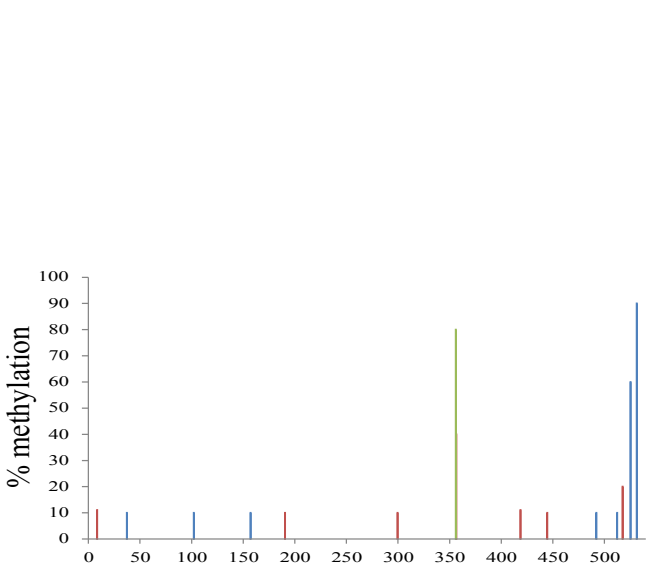

5B

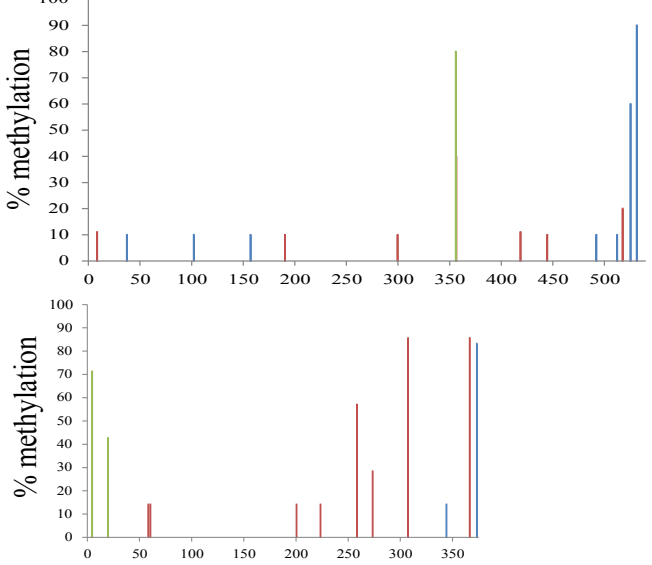

5D

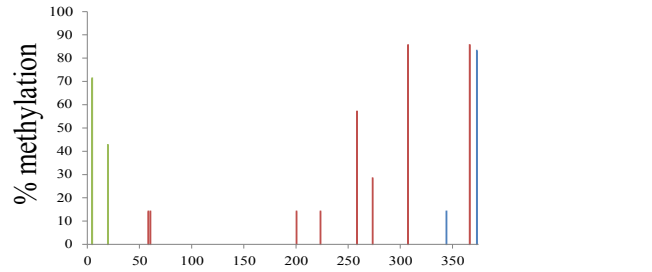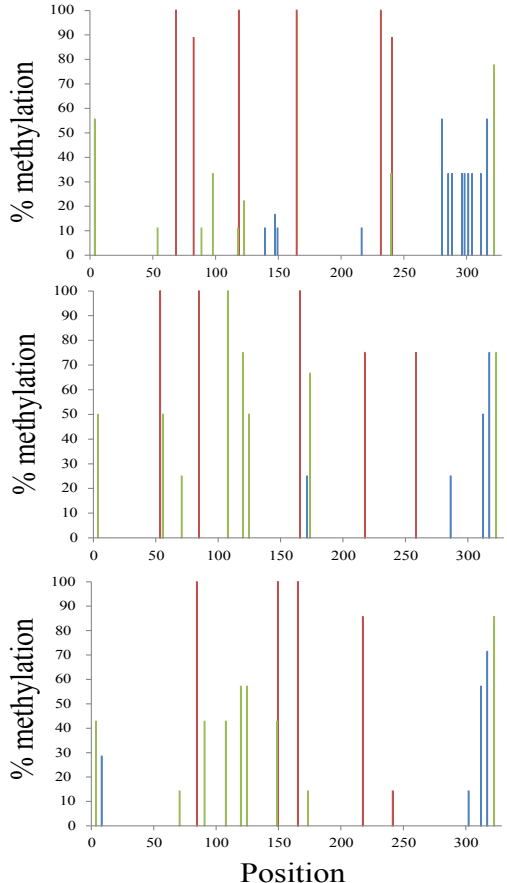

Supplement: Supplementary file 4 — Additional file 4: Bisulfite analysis of putative promoter region of homoeologous group 5. A) Frequencies of CG dinucleotides were computed every 50 bp of the putative promoter regions of homoeologous group 5. 5A (black), 5B (white) and 5D (grey). Black bars numbered from 1 to 4 highlight the two regions studied by bisulfite sequencing. B) Kismeth outputs of the percentage of methylated cytosines in CG (red), CHG green) and CHH (blue) context. (PDF 172 KB) [file 12864_2014_6631_MOESM4_ESM.pdf]

Additional file 5

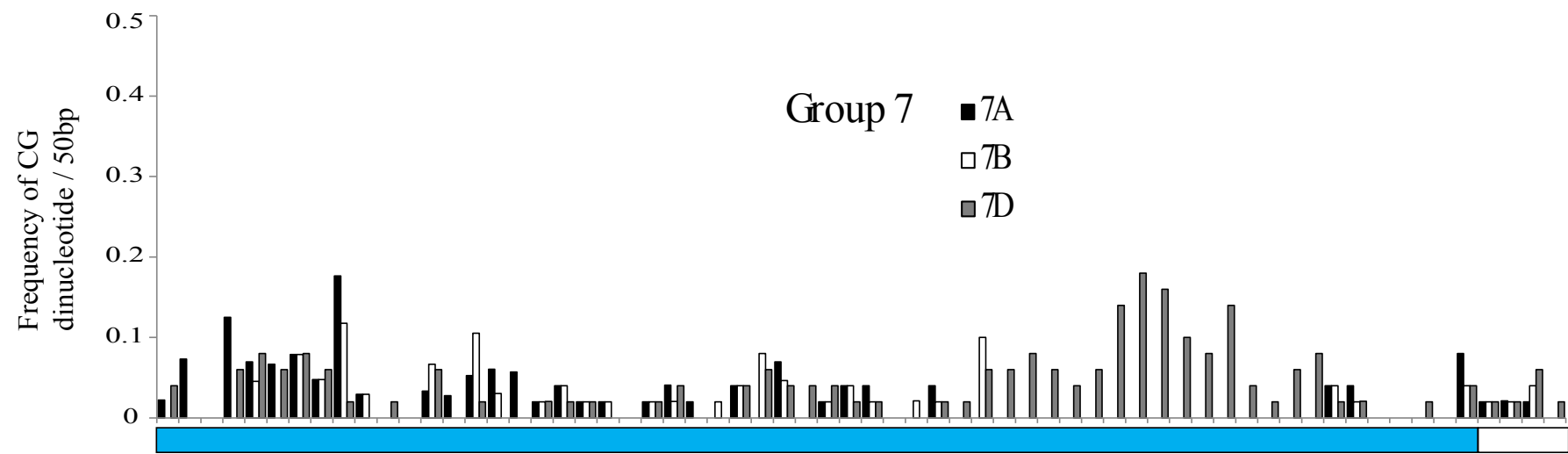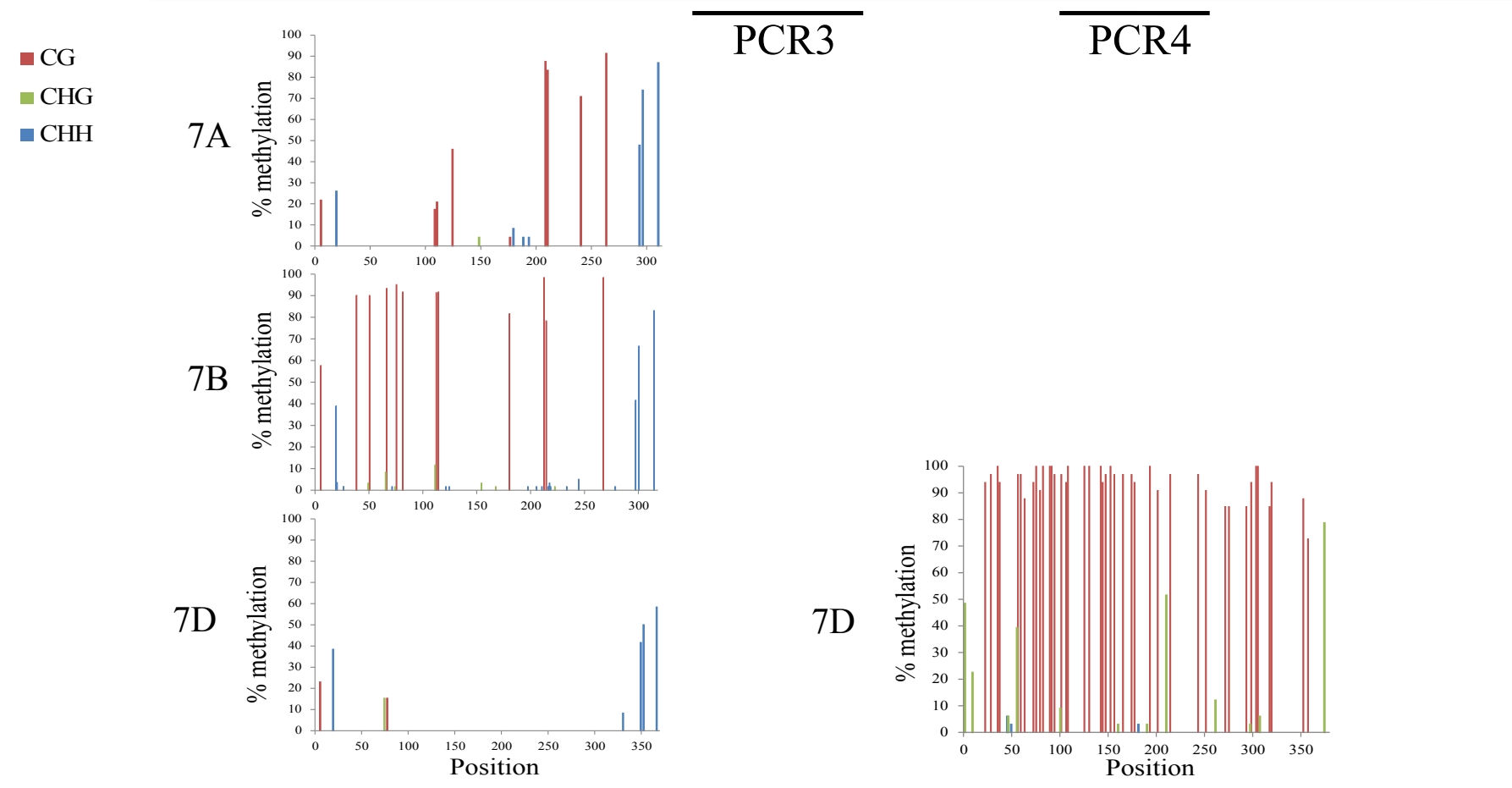

Supplement: Supplementary file 5 — Additional file 5: Bisulfite analysis of putative promoter region of homoeologous group 7. A) Frequencies of CG dinucleotides were computed every 50 bp of the putative promoter regions of homoeologous group 7. 7A (black), 7B (white) and 7D (grey). Black bars numbered from 1 to 4 highlight the two regions studied by bisulfite sequencing. B) Kismeth outputs of the percentage of methylated cytosines in CG (red), CHG green) and CHH (blue) context. (PDF 155 KB) [file 12864_2014_6631_MOESM5_ESM.pdf]

Additional file 9

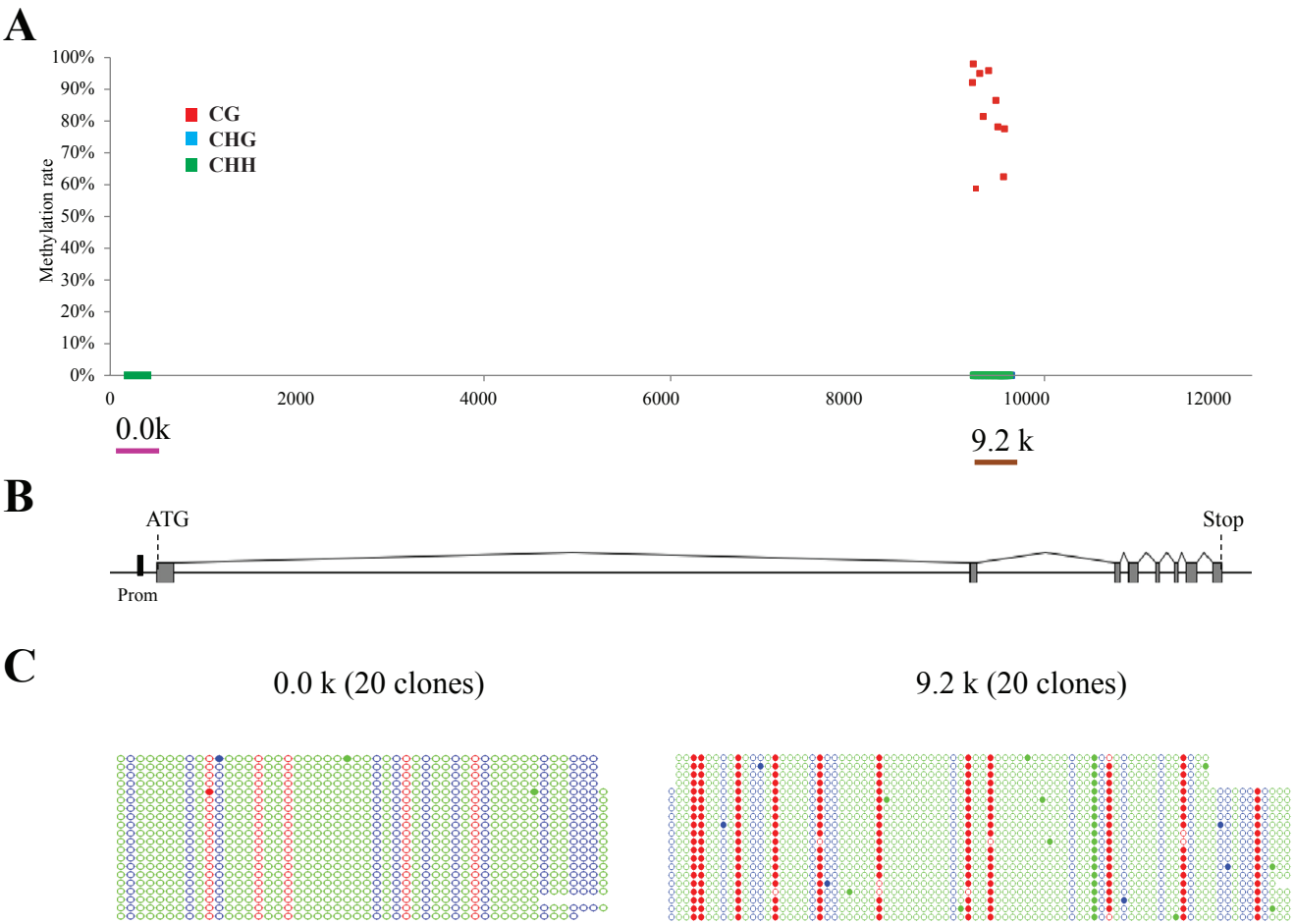

Supplement: Supplementary file 9 — Additional file 9: Controls in bisulfite experiments. A) Methylation rates at two VRN-A1 regions called 0.0 k and 9.2 k (adapted from [71]). B) Structure of the VRN-A1 gene. C) Typical results from bisulfite experiments for 0.0 k (no CG methylation) and 9.2 k (high CG methylation). (PDF 2 MB) [file 12864_2014_6631_MOESM9_ESM.pdf]
