# Supplementary material for: Evolutionary history of Methyltransferase 1 genes in hexaploid wheat
Source: BMC Genomics. 2014 Oct 23;15(1):922. doi: 10.1186/1471-2164-15-922 (PMC4223845; doi:10.1186/1471-2164-15-922)
Supplement: Supplementary file 6 — Additional file 6: Primers used in RT-PCR, RT-qPCR, mapping and bisulfite experiments. (PDF 20 KB) [file 12864_2014_6631_MOESM6_ESM.pdf]

# Additional file 6

| Gene | Homoeolog specificity | Methods | Forward primer (5' to 3') | reverse primer (5' to 3') | Remarks |
|---|---|---|---|---|---|
| MET1-group2 | 2BD | BS | TGATTTAYTTGGAGTGGTATGG | TTTCACAACTRTTTTTCTCTTCC | |
| | 2A | BS | GYTTTAYTTGGAGTGGTATGAA | TTCACAACTRTTTCTCTCTTCC | |
| | 2A | RT-PCR | GATAGGATTATCACAGTCCGC | GCAAGAGGGGGTGGCACA | |
| | 2B | RT-PCR | TGTGCCACCCCCTCTTGC | CAGTTCATGCCAGCCATGC | |
| | 2D | RT-PCR | GACCAGGATAGGATTATCACG | CCAGCCACGCACAATAAAGA | |
| | 2A | Q-RT-PCR | CAGTTTTCGGGCACCATTC | CCAGTCACGCACAATAGAGG | |
| | 2B | Q-RT-PCR | CAGTTTTCGGGCACCATTC | CAGTTCATGCCAGCCATGC | |
| | 2D | Q-RT-PCR | TGTGCCACCCCCTCTTGC | CCAGCCACGCACAATAAAGA | |
| | 2B | Mapping | GAAGGTGACCAAGTTCATGCTCA GTTCAGGGCTAGCAAACACG | GCTCCTGGAGAGATTCTTCCTGATT | marker 52, Population Renan x Recital, SNP =G |
| | 2B | Mapping | GAAGGTCGGAGTCAACGGATTTC AGTTCAGGGCTAGCAAACACT | TTCTTCCTGATTGGCCAGAACCGAT | marker 52, Population Renan x Recital, SNP =T |
| MET1-group5 | 5A | Mapping | GAAGGTGACCAAGTTCATGCTCA CTTGTGCCTCGGCAACTACAA | GGGAACAAAATCTACTGTCAACTATG CTT | marker 51, Population Renan x Recital, SNP =A |
| | 5A | Mapping | GAAGGTCGGAGTCAACGGATTAC TTGTGCCTCGGCAACTACAC | CTACTGTCAACTATGCTTCATTTTAG CCAA | marker 51, Population Renan x Recital, SNP =C |
| | 5B | Mapping | GAAGGTGACCAAGTTCATGCTAC ATTCTTAGTAAGCAACCATGTGT | CCTGTGGATAGCTTCACCTGTACTA | marker 35, Population Renan x Recital, SNP =A |
| | 5B | Mapping | GAAGGTGACCAAGTTCATGCTCC CAGATGAACGCCCTTTTCCTA | ATTCGGCATTCTAGAAGCAGGTTCAT AT | marker 43, Population Renan x Recital, SNP =A |
| | 5B | Mapping | GAAGGTGACCAAGTTCATGCTGA GGCGAGCCCATCTCCTG | GCAAAAGCGTATCGCCTCGAATCTT | marker 48, Population Renan x Recital, SNP =C |
| | 5B | Mapping | GAAGGTCGGAGTCAACGGATTAC ATTCTTAGTAAGCAACCATGTGC | GCTTCACCTGTACTATTCACATGATG AAA | marker 35, Population Renan x Recital, SNP =G |
| | 5B | Mapping | GAAGGTCGGAGTCAACGGATTCC AGATGAACGCCCTTTTCCTG | CAGGTTCATATGGCATCGCGCATT | marker 43, Population Renan x Recital, SNP =G |
| | 5B | Mapping | GAAGGTCGGAGTCAACGGATTGG AGGCGAGCCCATCTCCTA | GTATCGCCTCGAATCTTCTTCTGGAA | marker 48, Population Renan x Recital, SNP =T |
| | 5B | BS | TTTYATGYAAGGAAGTGGYAG | AACTCCTTTACCTTTTTRTCATC | |
| | 5B | BS | GTTTTTGYGGGAGGGATTTG | TCRRCCATCATCATTCATCC | |
| | 5B | BS | GGTGGGTTYTYAAYTTTTGAT | AACCACATRTCCATRTAAAACC | |
| | 5D | BS | TTYATGYAAGGAAGTGGYGG | TCCTTTCCCTTTTTRTTRTCAC | |
| | 5D | BS | GAAYTGTTGAAGGAYTATYTG | ARATCCTCRACCAAAACTCAT | |
| | 5ABD | BS | ATYTGAAYTTYTAATTAGGATGT | TCCCCTRTAAARTTTARAATCA | |
| | 5A | RT-PCR | TCGATGATCTGCATATCTATTC | ACTGCTTCGTTGGCTGGAG | |
| | 5B | RT-PCR | CAGGAAGATCAAGGGATTCC | ACTGCTTCGTTGGCTGGAG | |
| | 5D | RT-PCR | GTGCTAAAAATGATACTGTTATC | ACTGCTTCGTTGGCTGGAG | |
| | 7A | Mapping | GAAGGTGACCAAGTTCATGCTAT GCATCTTCGCTGAGTGATCACA | TGAGATTGAGCTCATTCATTTCTTTA GCTA | marker 20, population Brigadier x Alcedo, SNP =A |
| | 7A | Mapping | GAAGGTGACCAAGTTCATGCTAA CTCCACCGTTTGCCCTGTC | CAATCATGTGATGTACTGGYAGCTGA T | marker 22, Population Renan x Recital, SNP =G |
| | 7A | Mapping | GAAGGTGACCAAGTTCATGCTAG CACCTGCCTCTAAAATCCCAAAT | GCATTCCTTCATTCCCACATGTTCTA AA | marker 24, Population Renan x Recital, SNP =A |
| | 7A | Mapping | GAAGGTGACCAAGTTCATGCTGA TTGGCCTGAACCGATGCAC | GCATAGTATTTCCCTTCTGGTAGATT GAT | marker 25, Population Renan x Recital, SNP =C |
| | 7A | Mapping | GAAGGTGACCAAGTTCATGCTAG TCAGGGATACAATTGGTGATCTC | TACTATACACCTACCGGTATTGTTGG TTT | marker 26, Population Renan x Recital, SNP =C |
| | 7A | Mapping | GAAGGTGACCAAGTTCATGCTCA ACAGAACTAGCTGATTATGAATG T | TATGGGCCTTCTGACTGAAAAGATCA AAA | marker 30, Population Renan x Recital, SNP =A |
| | 7A | Mapping | GAAGGTGACCAAGTTCATGCTGG CGTAATTTGTACCTGAAATC | CGATGAAAGCTCCCTCTAACTTACAT AT | marker 34, population Brigadier x Alcedo, SNP =G |

| | | | | | |
|---|---|---|---|---|---|
| MET1-group7 | 7A | Mapping | GAAGGTGACCAAGTTCATGCTGATGCAATCATCAACATCACCACAC | TGACCATTTGCCTTTGCAGGGCAAT | marker 41, population Brigadier x Alcedo, SNP =C |
| | 7A | Mapping | GAAGGTCGGAGTCAACGGATTGCATCTTCGCTGAGTGATCACG | GCACCTTATGAGATTGAGCTCATTCATTT | marker 20, population Brigadier x Alcedo, SNP =G |
| | 7A | Mapping | GAAGGTCGGAGTCAACGGATTCAACTCCACCGTTTGCCCTGTA | GCTGTGCGTGCAGGTGAAGCTA | marker 22, Population Renan x Recital, SNP =T |
| | 7A | Mapping | GAAGGTCGGAGTCAACGGATTCACCTGCCTCTAAAATCCCAAAC | TCCCACATGTTCTAAACTTCTCTGCATTT | marker 24, Population Renan x Recital, SNP =G |
| | 7A | Mapping | GAAGGTCGGAGTCAACGGATTCTGATTGGCCTGAACCGATGCAT | TGATTTTCAGCTCAGGGCTGGAGAA | marker 25, Population Renan x Recital, SNP =T |
| | 7A | Mapping | GAAGGTCGGAGTCAACGGATTAGTCAGGGATACAATTGGTGATCTG | GGTATTGTTGGTTTGCTGGCACCAT | marker 26, Population Renan x Recital, SNP =G |
| | 7A | Mapping | GAAGGTCGGAGTCAACGGATTCAACAGAACTAGCTGATTATGAATGC | AAGACCGGTAACTACTTGCTGGCTT | marker 30, Population Renan x Recital, SNP =G |
| | 7A | Mapping | GAAGGTCGGAGTCAACGGATTGGCTGGCGTAATTTGTACCTGAAATA | CTCCCTCTAACTTACATATGGATCTCAAT | marker 34, population Brigadier x Alcedo, SNP =T |
| | 7A | Mapping | GAAGGTCGGAGTCAACGGATTGATGCAATCATCAACATCACCACAG | GCCTTTGCAGGGCAATAATGGACAA | marker 41, population Brigadier x Alcedo, SNP =G |
| | 7B | Mapping | GAAGGTGACCAAGTTCATGCTGCATTTATTTCAACAAATGCAGCGC | TGAAGAATTATCTGTCCATGCACCACAA | marker 04, population Brigadier x Alcedo, SNP =C |
| | 7B | Mapping | GAAGGTGACCAAGTTCATGCTATGCACCACAACATATCGTTCAACG | AATTTGAGAAAGGGCAGCCTGCATTTATT | marker 05, population Brigadier x Alcedo, SNP =C |
| | 7B | Mapping | GAAGGTGACCAAGTTCATGCTCACTTGCTTCGAATTCTAAACAGTCATT | CTGTCCTTGGCAATGCTGCAAATGAA | marker 06, population Brigadier x Alcedo, SNP =A |
| | 7B | Mapping | GAAGGTGACCAAGTTCATGCTTGCTCTCCTCTGTTTGTCCCTA | AAAAAATGCAGAGGTTTAGAACATGTGGAA | marker 09, population Brigadier x Alcedo, SNP =A |
| | 7B | Mapping | GAAGGTGACCAAGTTCATGCTGAGAGGGGAGGCGGCACG | ATTTCGCTGGCAACATCCAGAGCAA | marker 11, population Brigadier x Alcedo, SNP =C |
| | 7B | Mapping | GAAGGTCGGAGTCAACGGATTGCATTTATTTCAACAAATGCAGCGG | CTGTCCATGCACCACAACATATCGTT | marker 04, population Brigadier x Alcedo, SNP =G |
| | 7B | Mapping | GAAGGTCGGAGTCAACGGATTCATGCACCACAACATATCGTTCAACA | AAGGGCAGCCTGCATTTATTTCAACAAAT | marker 05, population Brigadier x Alcedo, SNP =T |
| | 7B | Mapping | GAAGGTCGGAGTCAACGGATTACTTGCTTCGAATTCTAAACAGTCATC | GCAATGCTGCAAATGAACGAGAGGTT | marker 06, population Brigadier x Alcedo, SNP =G |
| | 7B | Mapping | GAAGGTCGGAGTCAACGGATTGCTCTCCTCTGTTTGTCCCTG | GAGGTTTAGAACATGTGGAAATGAAGGAA | marker 09, population Brigadier x Alcedo, SNP =G |
| | 7B | Mapping | GAAGGTCGGAGTCAACGGATTGAGAGGGGAGGCGGCACC | TCCAGAGCAAGCACCGGCAGAT | marker 11, population Brigadier x Alcedo, SNP =G |
| | 7A | BS | GGGTYGAGTTTTGTATAAYTTA | AAARCCAAAACAAATAARAARAC | |
| | 7B | BS | GGGTYGAGTTTTGTATAAYYTA | AAARCCAAAACAAATAARAARAC | |
| | 7D | BS | GGGTYGAGTTTTGTATAAYATA | AAARCCAAAACAAATAARAARAC | |
| | 7D | BS | YTGGGTGGYAGAGAAGAATG | ACACARACTCCTCTAAATTCAC | |
| | 7A | RT-PCR | CATGCTTGTGGAGGAACTG | ATCTTTGCAAGAAGTCCAGTG | |
| | 7A | RT-PCR | CAGGATCATCACTGTTCGTG | AATGACGAGCCTTGTATCATG | |
| | 7D | RT-PCR | CAGGATCATCACTGTTCGTG | GGCGACCAGCCTGTATATAG | |
| | 7D | RT-PCR | GCTCAAGCAAGCCATCGAC | GGCGACCAGCCTGTATATAG | |
| *VRN-A1* | A | BS | AAATGATTTGGGGAAAGCAAAATC | GAGGGGTTTTTAAAGGGATCTGGCCC | 0.0 k fragment from Khan et al 2013 |
| | A | BS | GGAYAAAATTYTTGAAYGGTATGAG | TTRCTAARCCCTTCAAAAACTCA | 9.2 k fragment from Khan et al 2013 |
