# Supplementary material for: Evolutionary history of Methyltransferase 1 genes in hexaploid wheat
Source: BMC Genomics. 2014 Oct 23;15(1):922. doi: 10.1186/1471-2164-15-922 (PMC4223845; doi:10.1186/1471-2164-15-922)
Supplement: Supplementary file 7 — Additional file 7: Virtual physical map reconstruction at TaMET1 loci from micro-synteny data. Physical maps for Os and Bd, virtual physical map based on IWGSC surveys organized from rice and brachypodium orthologs. TaMET1 loci are highlighted in yellow. Note that two overlapping contigs were found at TaMET-5A1 indicating that these two IWGSC contigs were not assembled together in the course of the assembly process. (PDF 21 KB) [file 12864_2014_6631_MOESM7_ESM.pdf]

Additional file 7

CHROMOSOME ARM	IWGSC CONTIG	IWGSC ORDER	Os NAME	Os ORDER	Bd NAME	Bd ORDER	
5AL	5AL:2744104	1	Os03g58040.1	1	Bradi1g05680.1	1	
5AL	5AL:2732873	2	Os03g58050.1	2	Bradi1g05670.1	2	
5AL	5AL:2734524	3	Os03g58060.1	3	Bradi1g05660.1	3	
5AL	5AL:2732731	4	Os03g58070.1	4	Bradi1g05650.1	4	
5AL	5AL:2736066	5	Os03g58080.1	5	Bradi1g05630.1	5	
5AL	5AL:2695737	6	Os03g58090.1	6	Bradi1g05620.1	6	
5AL	5AL:2763495	7	Os03g58100.1	7	Bradi1g05610.1	7	
5AL	5AL:343725	8	Os03g58110.1	8	Bradi1g05600.1	8	
5AL	5AL:2686684	9	Os03g58120.1	9	Bradi1g05590.1	9	
5AL	5AL:353991	10	Os03g58130.1	10	Bradi1g05580.1	10	
5AL	5AL:2760754	11	Os03g58140.1	11	Bradi1g05570.1	11	
5AL	5AL:2760754	12	Os03g58150.1	12	Bradi1g05560.1	12	
5AL	5AL:683544	13	Os03g58160.1	13	Bradi1g05550.1	13	
5AL	5AL:2800470	14	Os03g58170.1	14			
5AL	5AL:2102481	15			Bradi1g05540.1	14	
5AL	5AL:2694483	16	Os03g58190.1	15	Bradi1g05520.1	15	
5AL	5AL:2691246	17	Os03g58204.1	16	Bradi1g05510.1	16	
5AL	5AL:1744178	18	Os03g58240.1	17	Bradi1g05490.1	17	
5AL	5AL:2741360	19	Os03g58250.1	18	Bradi1g05480.1	18	
5AL	5AL:2801238	20	Os03g58260.1	19			
5AL	5AL:2759119	21			Bradi1g05470.1	19	
5AL	5AL:1126042	22	Os03g58290.1	20	Bradi1g05460.1	20	
5AL	5AL:1126042	23	Os03g58300.1	21	Bradi1g05450.1	21	
5AL	5AL:1126042	24	Os03g58320.1	22			
5AL	5AL:2796056	25	Os03g58330.1	23	Bradi1g05430.1	22	
5AL	5AL:2802820	26	Os03g58340.1	24	Bradi1g05420.1	23	
5AL	5AL:2802820	27			Bradi1g05410.1	24	
5AL	5AL:2799595	28	Os03g58390.1	25	Bradi1g05390.1	25	
5AL	5AL:2806153	29	Os03g58400.1	26			TaMet-5A1
5AL	5AL:2672180	30			Bradi1g05380.1	26	TaMet-5A1
5AL	5AL:32991	31	Os03g58420.1	27	Bradi1g05350.1	27	
5AL	5AL:2734204	32	Os03g58430.1	28	Bradi1g05340.1	28	
5AL	5AL:1181641	33	Os03g58480.1	29	Bradi1g05320.1	29	
5AL	5AL:361545	34	Os03g58520.1	30	Bradi1g05260.1	30	
5AL	5AL:429134	35	Os03g58530.1	31	Bradi1g05250.1	31	
5AL	5AL:2376817	36	Os03g58540.1	32	Bradi1g05240.1	32	
5AL	5AL:2741481	37			Bradi1g05200.1	33	
5AL	5AL:1594861	38	Os03g58570.1	33	Bradi1g05190.1	34	
5AL	5AL:2789791	39	Os03g58590.1	34	Bradi1g05170.1	35	
5AL	5AL:2748163	40	Os03g58600.1	35	Bradi1g05160.1	36	
5AL	5AL:2352467	41	Os03g58620.1	36			
5AL	5AL:2809517	42	Os03g58630.1	37			
5AL	5AL:2803096	43			Bradi1g05150.1	37	
5AL	5AL:2798135	44			Bradi1g05140.1	38	
5AL	5AL:2806991	45			Bradi1g05130.1	39	
5AL	5AL:941218	46			Bradi1g05120.1	40	
5AL	5AL:2768199	47			Bradi1g05110.1	41	

5AL	5AL:2806991	48	Os03g58640.1	38			
5AL	5AL:598579	49	Os03g58670.1	39			
5AL	5AL:2782620	50	Os03g58700.1	40			
5AL	5AL:2809814	51	Os03g58710.1	41			
5AL	5AL:2768199	52	Os03g58720.1	42			
5AL	5AL:828164	53	Os03g58740.1	43	Bradi1g05100.1	42	
5AL	5AL:2353167	54			Bradi1g05080.1	43	
5AL	5AL:1685317	55	Os03g58780.1	44	Bradi1g05070.1	44	
5AL	5AL:2805920	56	Os03g58820.1	45	Bradi1g05060.1	45	
5AL	5AL:2796269	57	Os03g58830.1	46	Bradi1g05040.1	46	
5AL	5AL:1230154	58	Os03g58840.1	47	Bradi1g05030.1	47	
5AL	5AL:2741244	59	Os03g58850.1	48			
5AL	5AL:2809741	60			Bradi1g05020.1	48	
5AL	5AL:2682679	61	Os03g58870.1	49	Bradi1g05010.1	49	
5AL	5AL:986955	62	Os03g58890.1	50	Bradi1g05000.1	50	
5AL	5AL:1907283	63	Os03g58900.1	51	Bradi1g04990.1	51	
5AL	5AL:2783401	64	Os03g58910.2	52	Bradi1g04980.1	52	
5AL	5AL:2805442	65			Bradi1g04960.1	53	
5BL	5BL:10816777	1	Os03g58040.1	1	Bradi1g05680.1	1	
5BL	5BL:10797603	2	Os03g58050.1	2	Bradi1g05670.1	2	
5BL	5BL:10806818	3	Os03g58060.1	3	Bradi1g05660.1	3	
5BL	5BL:10721087	4	Os03g58070.1	4	Bradi1g05650.1	4	
5BL	5BL:10824935	5			Bradi1g05640.1	5	
5BL	5BL:8729501	6	Os03g58080.1	5	Bradi1g05630.1	6	
5BL	5BL:2853232	7	Os03g58090.1	6	Bradi1g05620.1	7	
5BL	5BL:10806811	8	Os03g58100.1	7	Bradi1g05610.1	8	
5BL	5BL:10806811	9	Os03g58110.1	8	Bradi1g05600.1	9	
5BL	5BL:10887964	10	Os03g58120.1	9	Bradi1g05590.1	10	
5BL	5BL:10845579	11	Os03g58130.1	10	Bradi1g05580.1	11	
5BL	5BL:10845579	12	Os03g58140.1	11	Bradi1g05570.1	12	
5BL	5BL:10845579	13	Os03g58150.1	12	Bradi1g05560.1	13	
5BL	5BL:10845579	14	Os03g58160.1	13	Bradi1g05550.1	14	
5BL	5BL:10920668	15	Os03g58170.1	14	Bradi1g05540.1	15	
5BL	5BL:10890955	16	Os03g58180.1	15	Bradi1g05530.1	16	
5BL	5BL:10899556	17	Os03g58190.1	16	Bradi1g05520.1	17	
5BL	5BL:10837461	18	Os03g58204.1	17	Bradi1g05510.1	18	
5BL	5BL:10826094	19	Os03g58230.1	18	Bradi1g05500.1	19	
5BL	5BL:10792150	20	Os03g58240.1	19	Bradi1g05490.1	20	
5BL	5BL:10189661	21	Os03g58250.1	20	Bradi1g05480.1	21	
5BL	5BL:10839350	22	Os03g58260.1	21	Bradi1g05470.1	22	
5BL	5BL:10899718	23	Os03g58290.1	22	Bradi1g05460.1	23	
5BL	5BL:10899718	24			Bradi1g05450.1	24	
5BL	5BL:10807820	25	Os03g58300.1	23			
5BL	5BL:10924991	26	Os03g58320.1	24			
5BL	5BL:10825701	27	Os03g58330.1	25			
5BL	5BL:10825701	28			Bradi1g05430.1	25	
5BL	5BL:10921140	29	Os03g58340.1	26	Bradi1g05420.1	26	
5BL	5BL:10921140	30	Os03g58350.1	27	Bradi1g05410.1	27	
5BL	5BL:10864426	31	Os03g58380.1	28			
5BL	5BL:7717076	32	Os03g58390.1	29	Bradi1g05390.1	28	
5BL	5BL:10925378	33	Os03g58400.1	30	Bradi1g05380.1	29	TaMet-5B1
5BL	5BL:10924332	34	Os03g58410.1	31	Bradi1g05370.1	30	

5BL	5BL:10867378	35	Os03g58420.1	32	Bradi1g05350.1	31
5BL	5BL:10795568	36	Os03g58430.1	33	Bradi1g05340.1	32
5BL	5BL:10822114	37			Bradi1g05330.1	33
5BL	5BL:10825924	38	Os03g58480.1	34	Bradi1g05320.1	34
5BL	5BL:10825924	39			Bradi1g05310.1	35
5BL	5BL:10893769	40	Os03g58500.1	35		
5BL	5BL:10814367	41			Bradi1g05280.1	36
5BL	5BL:10879097	42	Os03g58520.1	36	Bradi1g05260.1	37
5BL	5BL:10879097	43	Os03g58530.1	37	Bradi1g05250.1	38
5BL	5BL:10838104	44	Os03g58540.1	38	Bradi1g05240.1	39
5BL	5BL:10838104	45			Bradi1g05200.1	40
5BL	5BL:10840323	46	Os03g58570.1	39	Bradi1g05190.1	41
5BL	5BL:10844621	47	Os03g58580.1	40	Bradi1g05180.1	42
5BL	5BL:10914845	48	Os03g58590.1	41	Bradi1g05170.1	43
5BL	5BL:10914845	49			Bradi1g05160.1	44
5BL	5BL:10816505	50	Os03g58600.1	42		
5BL	5BL:10829640	51	Os03g58620.1	43		
5BL	5BL:10922830	52	Os03g58630.1	44	Bradi1g05150.1	45
5BL	5BL:10832199	53	Os03g58670.1	46		
5BL	5BL:10801521	54	Os03g58710.1	48	Bradi1g05140.1	46
5BL	5BL:10882913	55	Os03g58640.1	45	Bradi1g05130.1	47
5BL	5BL:10815386	56	Os03g58700.1	47	Bradi1g05120.1	48
5BL	5BL:10879461	57	Os03g58720.1	49	Bradi1g05110.1	49
5BL	5BL:10855303	58	Os03g58740.1	50	Bradi1g05100.1	50
5BL	5BL:10918676	59	Os03g58750.1	51	Bradi1g05090.1	51
5BL	5BL:10865573	60			Bradi1g05080.1	52
5BL	5BL:10789814	61	Os03g58780.1	52	Bradi1g05070.1	53
5BL	5BL:10735609	62	Os03g58790.1	53		
5BL	5BL:10735609	63	Os03g58800.1	54		
5BL	5BL:10787074	64	Os03g58810.1	55		
5BL	5BL:10840449	65	Os03g58820.1	56	Bradi1g05060.1	54
5BL	5BL:1283675	66	Os03g58830.1	57	Bradi1g05040.1	55
5BL	5BL:10867098	67	Os03g58840.1	58	Bradi1g05030.1	56
5BL	5BL:10824553	68	Os03g58850.1	59		
5BL	5BL:10893855	69			Bradi1g05020.1	57
5BL	5BL:10905571	70	Os03g58870.1	60	Bradi1g05010.1	58
5BL	5BL:10866215	71	Os03g58890.1	61	Bradi1g05000.1	59
5BL	5BL:10871750	72	Os03g58900.1	62	Bradi1g04990.1	60
5BL	5BL:10923312	73	Os03g58910.2	63	Bradi1g04980.1	61
5BL	5BL:10871750	74	Os03g58920.1	64	Bradi1g04960.1	62
5DL	5DL:4579940	1	Os03g58040.1	1	Bradi1g05680.1	1
5DL	5DL:4568611	2	Os03g58050.1	2	Bradi1g05670.1	2
5DL	5DL:4552307	3	Os03g58060.1	3	Bradi1g05660.1	3
5DL	5DL:4503194	4			Bradi1g05640.1	4
5DL	5DL:4532486	5	Os03g58080.1	4	Bradi1g05630.1	5
5DL	5DL:4532486	6	Os03g58090.1	5	Bradi1g05620.1	6
5DL	5DL:4603458	7	Os03g58100.1	6	Bradi1g05610.1	7
5DL	5DL:4603458	8	Os03g58110.1	7	Bradi1g05600.1	8
5DL	5DL:4541028	9	Os03g58120.1	8	Bradi1g05590.1	9
5DL	5DL:4513175	10	Os03g58130.1	9	Bradi1g05580.1	10
5DL	5DL:4513175	11	Os03g58140.1	10	Bradi1g05570.1	11
5DL	5DL:4513175	12	Os03g58150.1	11	Bradi1g05560.1	12

5DL	5DL:4513175	13	Os03g58160.1	12	Bradi1g05550.1	13	
5DL	5DL:4524567	14	Os03g58170.1	13	Bradi1g05540.1	14	
5DL	5DL:4500384	15	Os03g58190.1	14	Bradi1g05520.1	15	
5DL	5DL:857395	16	Os03g58204.1	15	Bradi1g05510.1	16	
5DL	5DL:4592014	17	Os03g58230.1	16	Bradi1g05500.1	17	
5DL	5DL:4603599	18	Os03g58240.1	17	Bradi1g05490.1	18	
5DL	5DL:4603599	19	Os03g58250.1	18	Bradi1g05480.1	19	
5DL	5DL:4600688	20	Os03g58260.1	19	Bradi1g05470.1	20	
5DL	5DL:4547569	21	Os03g58300.1	20			
5DL	5DL:4547569	22	Os03g58320.1	22	Bradi1g05460.1	21	
5DL	5DL:4496471	23	Os03g58290.1	23	Bradi1g05450.1	22	
5DL	5DL:4515833	24	Os03g58330.1	21	Bradi1g05430.1	23	
5DL	5DL:4490001	25	Os03g58340.1	24	Bradi1g05420.1	24	
5DL	5DL:4490001	26	Os03g58350.1	25	Bradi1g05410.1	25	
5DL	5DL:3393307	27	Os03g58390.1	26	Bradi1g05390.1	26	
5DL	5DL:4608210	28	Os03g58400.1	27	Bradi1g05380.1	27	TaMet-5D1
5DL	5DL:4567022	29	Os03g58410.1	28	Bradi1g05370.1	28	
5DL	5DL:4566006	30	Os03g58420.1	29	Bradi1g05350.1	29	
5DL	5DL:4524187	31	Os03g58430.1	30	Bradi1g05340.1	30	
5DL	5DL:4556738	32			Bradi1g05330.1	31	
5DL	5DL:2336334	33	Os03g58480.1	33	Bradi1g05320.1	32	
5DL	5DL:2910741	34	Os03g58500.1	34			
5DL	5DL:4571477	35			Bradi1g05290.1	33	
5DL	5DL:4571477	36			Bradi1g05270.1	34	
5DL	5DL:1047956	37			Bradi1g05280.1	35	
5DL	5DL:4608660	38	Os03g58520.1	35	Bradi1g05260.1	36	
5DL	5DL:4473836	39	Os03g58530.1	36	Bradi1g05250.1	37	
5DL	5DL:4495237	40	Os03g58540.1	37	Bradi1g05240.1	38	
5DL	5DL:4495237	41			Bradi1g05200.1	39	
5DL	5DL:4572451	42	Os03g58570.1	38	Bradi1g05190.1	40	
5DL	5DL:4532064	43	Os03g58580.1	39	Bradi1g05180.1	41	
5DL	5DL:4532779	44	Os03g58590.1	40	Bradi1g05170.1	42	
5DL	5DL:4557608	45	Os03g58600.1	41	Bradi1g05160.1	43	
5DL	5DL:4546142	46	Os03g58620.1	42			
5DL	5DL:4571419	47	Os03g58630.1	43	Bradi1g05150.1	44	
5DL	5DL:4571419	48	Os03g58640.1	44	Bradi1g05140.1	45	
5DL	5DL:4571419	49			Bradi1g05130.1	46	
5DL	5DL:4523323	50	Os03g58700.1	45	Bradi1g05120.1	47	
5DL	5DL:4608197	51	Os03g58710.1	46			
5DL	5DL:4541544	52	Os03g58720.1	47	Bradi1g05110.1	48	
5DL	5DL:4517824	53	Os03g58740.1	48	Bradi1g05100.1	49	
5DL	5DL:4603756	54	Os03g58780.1	49	Bradi1g05080.1	50	
5DL	5DL:4489274	55	Os03g58790.1	50			
5DL	5DL:4517163	56	Os03g58800.1	51			
5DL	5DL:4585906	57	Os03g58810.1	52			
5DL	5DL:4509470	58	Os03g58820.1	53			
5DL	5DL:4603756	59			Bradi1g05070.1	51	
5DL	5DL:4509470	60			Bradi1g05060.1	52	
5DL	5DL:4606041	61			Bradi1g05050.1	53	
5DL	5DL:4489431	62	Os03g58830.1	54	Bradi1g05040.1	54	
5DL	5DL:4572971	63	Os03g58840.1	55	Bradi1g05030.1	55	
5DL	5DL:4551533	64			Bradi1g05020.1	56	

5DL	5DL:4565592	65	Os03g58850.1	56			
5DL	5DL:4599310	66	Os03g58870.1	57			
5DL	5DL:4599310	67	Os03g58890.1	58			
5DL	5DL:4570311	68	Os03g58900.1	59			
5DL	5DL:4496706	69	Os03g58910.2	60			
5DL	5DL:4557991	70	Os03g58920.1	61			
7AL	7AL:4500764	1	Os03g58040.1	1	Bradi1g05680.1	1	
7AL	7AL:4535568	2	Os03g58050.1	2	Bradi1g05670.1	2	
7AL	7AL:4532056	3	Os03g58400.1	3	Bradi1g05380.1	3	TaMet-7A1
7AL	7AL:4441897	4			Bradi1g05360.1	4	
7AL	7AL:4534795	5	Os03g58430.1	5	Bradi1g05340.1	5	
7AL	7AL:1206673	6			Bradi1g05210.1	6	
7AL	7AL:4452921	7	Os03g58570.1	7	Bradi1g05190.1	7	
7AL	7AL:4510505	8	Os03g58580.1	8	Bradi1g05180.1	8	
7AL	7AL:4492195	9	Os03g58630.1	9	Bradi1g05150.1	9	
7AL	7AL:4492195	10			Bradi1g05140.1	10	
7AL	7AL:4483901	11	Os03g58670.1	11			
7AL	7AL:4472017	12	Os03g58790.1	12			
7AL	7AL:4472017	13	Os03g58800.1	13			
7AL	7AL:4472191	14	Os03g58810.1	14			
7AL	7AL:4472620	15	Os03g58830.1	15	Bradi1g05040.1	11	
7BL	7BL:6701310	1	Os03g58040.1	1	Bradi1g05680.1	1	
7BL	7BL:6694785	2	Os03g58050.1	2	Bradi1g05670.1	2	
7BL	7BL:6665951	3	Os03g58160.1	3	Bradi1g05550.1	3	
7BL	7BL:6682174	4	Os03g58400.1	4	Bradi1g05380.1	4	TaMet-7B1
7BL	7BL:279551	5	Os03g58670.1	6			
7BL	7BL:6743224	6	Os03g58790.1	7			
7BL	7BL:6674971	7	Os03g58800.1	8			
7BL	7BL:6697122	8			Bradi1g05360.1	5	
7BL	7BL:6501267	9			Bradi1g05350.1	6	
7BL	7BL:6710382	10			Bradi1g05290.1	7	
7BL	7BL:6632036	11			Bradi1g05280.1	8	
7BL	7BL:6710382	12			Bradi1g05270.1	9	
7BL	7BL:6745812	13			Bradi1g05210.1	10	
7BL	7BL:6689829	14	Os03g58570.1	5	Bradi1g05190.1	11	
7BL	7BL:6712874	15	Os03g58830.1	9	Bradi1g05040.1	12	
7DL	7DL:3378869	1	Os03g58040.1	1	Bradi1g05680.1	1	
7DL	7DL:3359841	2	Os03g58050.1	2	Bradi1g05670.1	2	
7DL	7DL:3344608	3			Bradi1g05570.1	3	
7DL	7DL:3392185	4	Os03g58400.1	3	Bradi1g05380.1	4	TaMet-7D1
7DL	7DL:3392185	5	Os03g58410.1	4	Bradi1g05370.1	5	
7DL	7DL:3374548	6			Bradi1g05360.1	6	
7DL	7DL:2581233	7			Bradi1g05350.1	7	
7DL	7DL:3368356	8			Bradi1g05210.1	8	
7DL	7DL:3341999	9	Os03g58570.1	5	Bradi1g05190.1	9	
7DL	7DL:3361750	10	Os03g58580.1	6	Bradi1g05180.1	10	
7DL	7DL:3366313	11	Os03g58670.1	7			
7DL	7DL:3393412	12	Os03g58790.1	8			
7DL	7DL:3393412	13	Os03g58800.1	9			
7DL	7DL:3387834	14	Os03g58810.1	10			
7DL	7DL:3392066	15			Bradi1g05050.1	11	
