# Supplementary material for: Evolutionary history of Methyltransferase 1 genes in hexaploid wheat
Source: BMC Genomics. 2014 Oct 23;15(1):922. doi: 10.1186/1471-2164-15-922 (PMC4223845; doi:10.1186/1471-2164-15-922)
Supplement: Supplementary file 8 — Additional file 8: RNA-seq samples used in this study. (PDF 33 KB) [file 12864_2014_6631_MOESM8_ESM.pdf]

Additional file 8

Stage	Wheat growth stage	Feekes scale	Zadoks scale	Leaves	Root	Stem	Spike	Grain
Seedling	First leaf through coleoptile	1	10	x	x			
Three leaves	3 leaves unfolded		13		x			
Three tillers	Main shoot and 3 tillers		23	x				
Spike at 1 cm	Pseudostem erection	5	30			x		
Two nodes	2nd detectable node	7	32			x	x	
Meiosis	Flag leaf ligule and collar visible	9	39		x		x	
Anthesis	1/2 of flowering complete		65			x	x	
2 DAAs (50°C.days)	Kernel (caryopsis) watery ripe		71	x				x
14 DAAs (350°C.days)	Medium Milk		75					x
30 DAAs (700°C.days)	Soft dough		85					x
